# Multi‐scale Engineered Vasculature and Hierarchical Porosity via Volumetric Bioprinting‐Guided Photopolymerization‐Induced Phase Separation

**DOI:** 10.1002/adma.202521171

**Published:** 2025-12-09

**Authors:** Oksana Y. Dudaryeva, Maj‐Britt Buchholz, Gabriel Größbacher, Sofia Amaral, Sammy Florczak, Camille Bonhomme, Alvaro Rojo Ferrer, Mark W. Tibbitt, Riccardo Levato

**Affiliations:** ^1^ Department of Orthopaedics University Medical Center Utrecht Utrecht University Utrecht 3584 CX The Netherlands; ^2^ Princess Máxima Center for Pediatric Oncology Utrecht 3584CS The Netherlands; ^3^ Macromolecular Engineering Laboratory Department of Mechanical and Process Engineering ETH Zurich Zurich 8092 Switzerland; ^4^ Department of Clinical Science Faculty of Veterinary Medicine Utrecht University Utrecht 3584CT The Netherlands

**Keywords:** 3D cell culture, bioprinting, controlled porosity, hydrogels, perfusion

## Abstract

Vascularization remains a major challenge in hydrogel‐based engineered tissues due to the inherent nano‐scale porosity of common synthetic and natural biomaterials. Critically, the confinement imposed by nanoscale networks inhibits blood vessels outgrowth, required for oxygen and nutrient delivery. Despite advancements in the biofabrication of small channels (0.1–1 mm), achieving vascularization (with capillaries down to 10 µm) throughout cm‐scale bioprinted constructs remains a critical bottleneck. Herein, phase separating is integrated, cell–interactive gelatin–norbornene hydrogels with volumetric bioprinting to generate architecturally defined centimeter‐scale constructs with 0.1–1mm scale printed channels and interpenetrating micron‐scale porosity. This novel approach produced freeform construct designs with light‐controllable micron‐scale and hierarchical porosity. Importantly, this porosity enabled endothelial cell infiltration and microvessel outgrowth deep into the engineered tissue. Vascular structures formed in the pore spaces with feature sizes on the scale of capillaries (<10 µm), crucial to provide oxygen and nutrients to all regions of the hydrogel. The networks remained stable for over 14 days, outperforming classical nanoporous biomaterials. Vascular networks are perfusable in this custom‐made bioreactor system and exhibited extended vessel outgrowth under perfused culture conditions. These complex hydrogel‐based constructs with engineered multi‐scale vascular networks have potential for generating actively perfusable advanced tissue models.

## Graphical abstract

Vascularization in bioprinted hydrogels is limited by nanoscale porosity in hydrogel bionks. Volumetric bioprinting of gelatin–norbornene phase‐separating hydrogels permits to create centimeter‐scale constructs featuring interconnected multi‐scale porosity. Light dose gradients result in spatially controlled pore dimensions. Capillary‐scale vessels are grown from bioprinted endothelial cells, and printed channels can be perfused, enabling the biofabrication of perfusable tissue models.

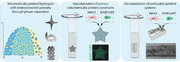

## 1 Introduction

Blood vessels form complex networks that interpenetrate and perfuse tissues and organs, providing critical nutrients and oxygen while removing metabolic waste. In addition, vascular networks are dynamic and rapidly adapt to physiologic changes to meet the changing metabolic demands of tissues. In this context, they are essential for maintaining tissue homeostasis and supporting cellular function. Vascular disorders, such as atherosclerotic cardiovascular disease, that impair this crucial function remain the leading cause of mortality across all developed societies and exacerbate comorbidities, including hypertension, hyperlipidemia, and pain disorders.^[^ ^1^ ^]^ Restriction of blood supply as a consequence of vascular disorders leads to ischaemia, nutrient depletion, and waste accumulation, ultimately causing tissue or organ necrosis or death.

Engineering vasculature in vitro is therefore essential for biological investigation of vascular function and for the fabrication of functional tissue replacement in regenerative medicine. However, the creation of large, fully vascularized, clinically relevant vascularized constructs remains a major challenge. Several strategies have been explored to produce viable and functioning vasculature in vitro in 3D engineered tissues. Often hydrogels based on natural extracellular matrix (ECM) components, such as collagen, or fibrin are used to generate in vitro vasculature with limited control of the vascular architecture.^[^ ^2^, ^3^ ^]^ To generate topologically defined vascular features within natural hydrogels, microfabrication or advanced sacrificial molding strategies have been applied.^[^ ^4^, ^5^ ^]^ While effective at small scales, this strategy is limited mainly to fabrication of enclosed microfluidic chips, which are necessary to maintain the mechanical integrity of the natural hydrogels during long‐term culture. The difficulties in handling these soft hydrogels and their limited stability impede their use in the broader context of biofabrication, especially for producing large‐scale constructs with complex shapes and architectural cues, i.e., tissue‐specific geometries with multi‐scale branching or interconnected loops that mimic native vasculature.

In this context, the ability to generate implantable vascularized hydrogel constructs with appropriate stability and structural integrity while capturing shape‐complexity is desirable. Photocurable synthetic hydrogels are often used in biofabrication as they offer appropriate stability and structural integrity and enable tailored structural cues due to their spatio‐selective photocrosslinking. However, synthetic photocrosslinkable biomaterials exhibit challenges due to their inherent nanoporosity, stiffness, or limited viscoelasticity which often impede effective outgrowth of 3D vascular structures.^[^ ^6^, ^7^ ^]^ To overcome these limitations and facilitate effective cell outgrowth in photocrosslinkable hydrogels they have been designed with inclusion of porogens, removable particles that are leached out after hydrogel crosslinking often resulting in poorly defined and non‐interconnected pores.^[^ ^8^, ^9^ ^]^ Other approaches rely on aqueous two‐phase emulsions (ATPE), in which phase separation occurs spontaneously upon mixing immiscible polymer solutions such as polyethyleneglycol (PEG) and dextran. This spontaneous demixing affords little temporal or spatial control, making it difficult to precisely tune pore size, distribution, or interconnectivity.^[^ ^10^, ^11^ ^]^

Alongside biomaterial developments, light‐based bioprinting techniques, including digital light processing (DLP) and volumetric bioprinting (VBP),^[^ ^8^, ^9^ ^]^ have emerged as effective methods to create intricate networks of 3D channels using photoresponsive hydrogels that recapitulate the complex anatomical architecture of native vasculature.^[^ ^11^, ^12^ ^]^ These permissive channels can be populated with living cells outgrowing into vascular networks, accelerating progress toward the in vitro fabrication of large‐scale vasculature. The bioprinting techniques, such as DLP and VBP, can effectively pattern large channels to establish static vessels in the 0.05–1 mm range, recapitulating the hierarchical and relatively static architecture of large‐scale vasculature. The dimensions of the microvasculature (<10 µm) are more challenging to capture with these techniques especially in the context of their randomly organized, highly dynamic nature that is capable of continuous sprouting and remodeling in response to external cues.^[^ ^12^, ^13^ ^]^ One strategy that overcomes the resolution limit is the use of multiphoton lithography, which enables sub‐micron feature generation but requires impractical amounts of time to construct large, clinically‐relevant microvascular networks, accommodating their dynamic remodelling.^[^ ^15^, ^16^ ^]^

Therefore, advanced biofabrication techniques that facilitate rapid formation of dense networks of capillaries less than 10 µm in size are needed to enable nutrient exchange across large volume engineered tissue models or to avoid rejection of biomaterial‐based implants. Ideally, such approaches would also facilitate the interface between channels and vessels (0.1–1 mm) and the generated microvasculature, enabling facile perfusion of the entire construct. Such multi‐scale engineered vascular networks would address the open challenge of microvascularization of in vitro tissue models, which is a determining factor for their success in disease modeling and drug screening pipelines.

Recent strategies for achieving superior porosity control, including dimensions within the microcapillary range, have relied on polymerization‐induced phase separation (PIPS) in PEG‐ and polyvynylalcohol (PVA)‐based systems, but so far have demonstrated only proof‐of‐concept bioprinting feasibility.^[^ ^14^, ^15^ ^]^ In this work, we generated bioprinted constructs from materials that enable vascularization of cm‐scale in vitro tissue models. We fabricated materials permissive for cell infiltration and vasculature outgrowth using photocontrolled phase‐separating bioinks based on the commonly used biomaterials: gelatin and PEG. Building on earlier PIPS‐based approaches we integrated cell‐interactive gelatin with volumetric bioprinting to achieve porosity control across large cm‐scale architecturally complex bioprinted structures. The integration of gelatin is essential for large‐scale bioprinting applications because no other commonly available biomaterial combines intrinsic cell bioactivity with cost‐effectiveness at this scale. The presence of cell‐adhesive motifs (arginine, glycine, aspartic acid RGD sequences) and enzymatically degradable sites [matrix metalloproteinase (MMP)‐sensitive domains] eliminates the need for challenging and expensive chemical modifications like peptide conjugation. Utilization of these materials for volumetric bioprinting enabled generation of bioprinted constructs with multi‐modal porosities of a controllable size. Importantly, this enabled us to generate prints with large pores or channels (mm‐scale) interconnected with micron‐sized channels (<10 µm) within a single porous construct. To our knowledge, this is one of the few strategies that are capable of providing control across multiple scales: shape architecture control at the millimeter‐scale combined with control over the intrinsic porosity at micron‐scale. These multi‐scale constructs were populated with vascular endothelial cells to create endothelial structures down to the microcapillary range. Generation of microvasculature in vitro using macroporous hydrogels will expedite the production of large vascularized in vitro models with tunable architectures. Such models will likely enable controlled parametric studies that will fundamentally enhance our understanding of angiogenesis in the course of disorders and accelerate production of vascularized engineered tissues and organs.

## 2 Results and Discussion

### 2.1 Engineering Phase‐Separating Bioinks for Volumetric Bioprinting

**Figure 1:**
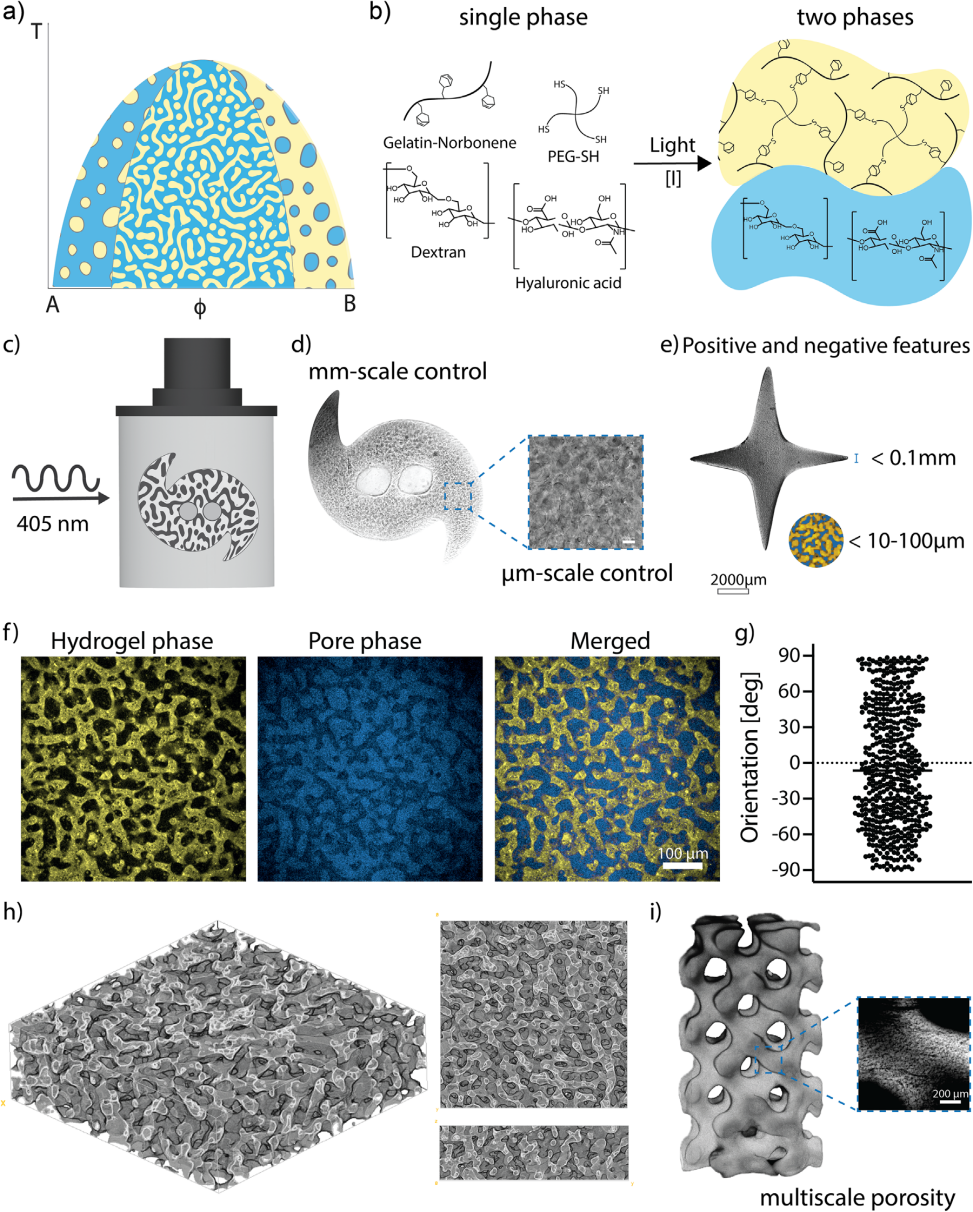
Formation of porous hydrogels from gelatin and PEG, and polysaccharides. a) In a multi‐component system, the initially miscible macromolecules [gelatin (A, yellow) and polysaccharides (B, blue)] become immiscible upon photocrosslinking of one of the components. Growth of the molecular weight induces phase separation and pore formation, either through binodal (formation of spheres) or spinodal decomposition (interconnected porosity) mechanisms depending on the composition (φ) and temperature (T) of the mixture. b) Our phase‐separated system is based on photocrosslinkable GelNb–PEG‐SH macromolecules and an excluded phase containing the polysaccharides dextran and HA. c) The phase‐separating materials can be volumetrically printed generating complex 3D objects with intrinsic interconnected porosity. d) Printing macroporous hydrogels provides control over mm‐ and micron‐scale features. Transmitted light image. e) The large‐scale features can be printed with a submillimeter resolution, the microscale features are printed with varying resolution in the range of 5–100 µm. Cropped transmitted light image. f) Photocrosslinking of Gelatin‐NB/PEG‐SH (gel‐phase; yellow) in presence of dextran/HA (excluded phase; blue) crosslinked at 5.6 mW cm^−2^ yielded hydrogel materials with interconnected perfusable porosity, demonstrated by perfusion of the pore phase with high Mw dextran‐FITC (Mw = 500 kDa). g) The spinodal porosity is isotropic in all directions as shown by the quantitative analysis of the pore orientation. h) 3D reconstruction of the phase‐separated hydrogel reveals a continuous porous network throughout the volume. The image was reconstructed from confocal z‐stacks and rendered using the Volume Viewer plugin in Fiji. i) Multiscale porous gyroid architectures were printed at *I*_av_ = 6 mW cm^−2^ featuring designed macroporosity (≈1 mm) and intrinsic, phase separation‐derived micron‐scale porosity (*n* = 3; 13.5 ± 2 µm). Scale bar = 200 µm.

Previously, we and others have established macroporous hydrogel biomaterials through PIPS.^[^ ^14^, ^15^ ^]^^16‐153^ In PIPS photocrosslinking of one component in an initially miscible multicomponent mixture (e.g., gelatin with polysaccharides) increases its molecular weight, driving phase separation and pore formation. Depending on composition (φ) and temperature (T), phase separation proceeds via binodal decomposition (discrete spherical domains) or spinodal decomposition (interconnected porous networks). Because the process is coupled to light‐controlled polymerization kinetics, the evolution of microstructure can be controlled and arrested at will, enabling exceptional control over pore size and architecture (**Figure** **1**a).^[^ ^14^ ^]^ We formulated phase separating bioresins based on gelatin and PEG polymers with controllable interconnected porosity based on thiol–ene chemistry to facilitate photopolymerization‐induced phase separation during crosslinking. The formulation consisted of gel forming components [norbornene‐functionalized gelatin (GelNb) and thiol‐functionalized 4‐arm poly(ethylene glycol) (PEG‐SH; 10kDa)]; an excluding phase [dextran (MW ≈ 100 kDa)]; and a viscosity modifier [hyaluronic acid (HA; *M*_n_ ≈ 2 MDa)] (Figure 1b).

In regenerative medicine and tissue engineering, there remains a major challenge in capturing the hierarchical architecture of living tissues, which spans from subcellular‐sized features to large anatomical structures. In this work, we sought to apply this concept in the design of biofunctional macroporous bioinks suitable for volumetric bioprinting (VBP) to engineer constructs with controlled millimeter‐scale features and micron‐scale interconnected porosity (Figure 1c,d). This allowed us to generate structures via VBP with features with size below 0.1 mm and micron scale porosity in the ranges of 5–100 µm. Crosslinking of GelNb–PEG‐SH hydrogels in the presence of dextran and HA generated materials with interconnected and perfusable porosity as demonstrated by perfusion of the porous space with large molecular weight Dextran‐FITC (500 kDa; Figure 1f). Generating well‐defined porosity in gelatin‐based systems was challenging due to temperature‐dependent physical interactions between gelatin macromolecules. At room temperature gelatin chains exhibit physical interactions leading to uncontrolled aggregation of the chains which could trigger phase separation before polymerization and affect the architecture and pore size of the final structures (Figure S1, Supporting Information). Maintaining a constant temperature was important for reproducible results. We maintained the temperatures above 37 °C (38–45 °C) during crosslinking to yield bicontinuous network structures formed via spinodal decomposition. The porosity was isotropically distributed throughout the hydrogel volume as shown by porosity orientation (Figure 1g,h). The phase‐separated inks were used to generate constructs with multi‐scale porosity, such as a gyroid that was designed with large‐scale printed porosity of 1 mm and exhibited small‐scale macroporosity of 13.5 ± 2 µm pores driven through phase‐separation, which can potentially enable enhanced mass transport and cell infiltration (Figure 1i; Figure S2, Supporting Information).

### 2.2 Controlling Porosity with Formulation, Irradiation Intensity, and Volumetric Light Fields

**Figure 2:**
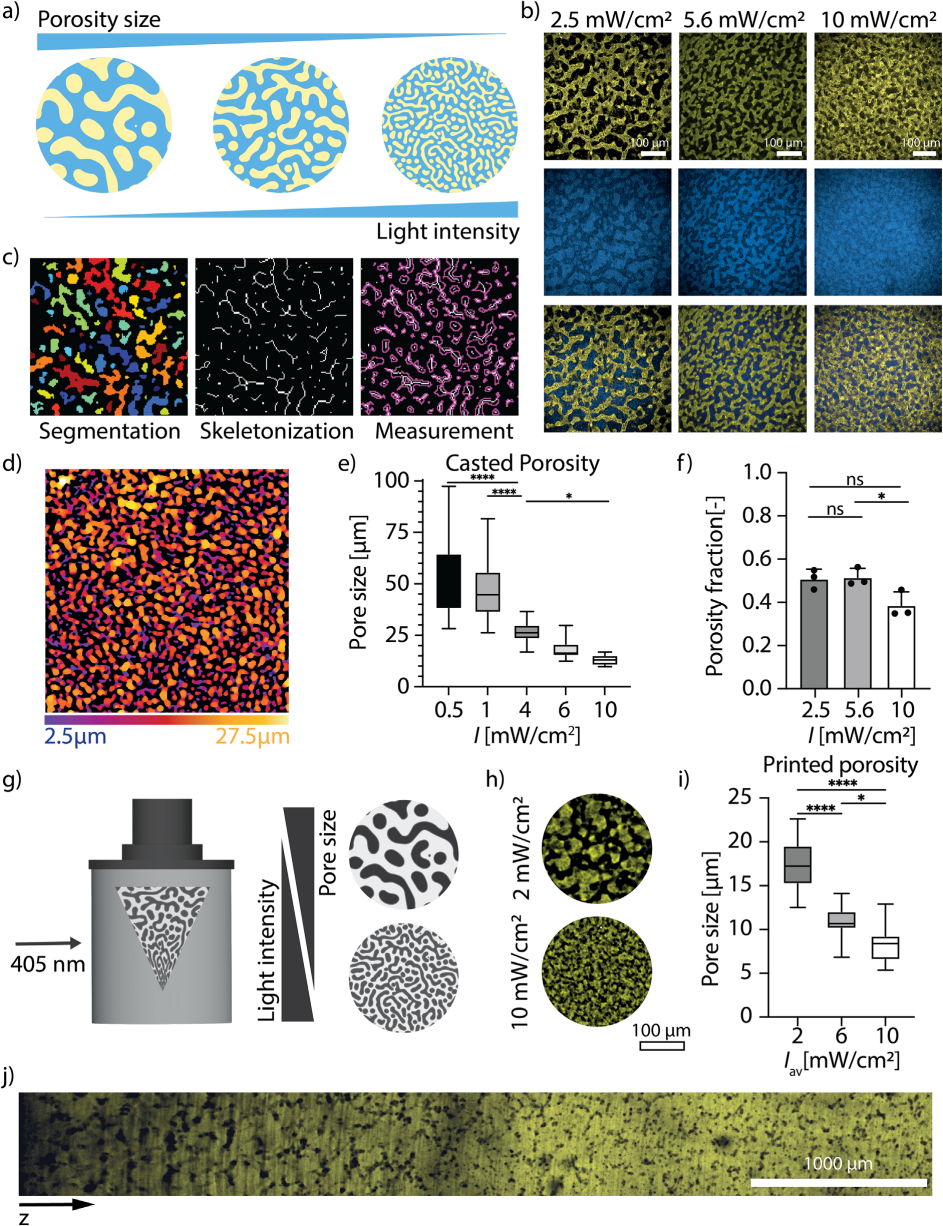
Controlling material porosity using light intensity. a) The size of porous features in photopolymerizable gelatin/PEG systems can be controlled using irradiation intensity, which governs polymerization kinetics. The final porosity increases at decreasing polymerization intensity. b) Confocal images depicting the porosity of the casted hydrogels, with pore size decreasing with increasing intensity. c) Representation of the porosity analysis pipeline. In order to characterize the pore sizes of the phase‐separated systems the 3D micrograph stack was first segmented within ImageJ and then processed in using the BoneJ plugin. This also enabled for a skeletonized depiction of the voids within the construct. d) The image presents a color‐coded map of pore size distribution within the hydrogel matrix, where each color corresponds to a specific pore width. The porosity ranged between 2.5 and 27.5 µm for hydrogels polymerized at 2.5 mW cm^−2^. e) The porosity within the casted systems has been controlled between 13.0 ± 2.4 to 52.9 ± 18.2 µm for 0.5 and 10 mW cm^−2^ respectively (*n* = 3). Box plots represent the interquartile range (25th–75th percentile), medians, and whiskers show the full data range (minimum to maximum). Pore size decreases significantly with increasing light intensity (* = p < 0.05, **** = p < 0.0001; one‐way ANOVA test). f) The fraction of porosity varied between 0.38 ± 0.06 for 10 mW cm^−2^ and 0.51 ± 0.04 for 5.6 mW cm^−2^ polymerization intensities (*n* = 3). Values are reported as mean and std. dev. (* = p < 0.05, ns = not significant). g) Volumetric printing enables generation of objects with varying porosity or with porosity gradients using volumetric light fields, either through printing objects at varying light intensity or applying the light fields with gradient intensity. h) The porosity of the printed objects was controlled by printing objects at different light intensities. i) The porosity was controlled between 17.5 ± 3.2 µm at *I_av_* = 2 mW cm^−2^ and 8.3 ± 2.0 µm at *I_av_* = 10 mW cm^−2^ respectively (*n* = 3). Box plots represent the interquartile range (25th–75th percentile), medians, and whiskers show the full data range (minimum to maximum). (* = p < 0.05, **** = p < 0.0001; one‐way ANOVA test). j) A cylindrical object was printed with the light field with gradient *I_av_* decreasing along the cylinder's z‐axis from 8 to 0.8 mW cm^−2^, generating a porosity gradient with the porosity sizes increasing along the decreasing *I_av_*. The tile scan image was taken along the z‐axis of the cylinder. Scale bar = 1000 µm.

To understand the design parameters for photopolymerization‐induced phase separation in this system, we first assessed how the formulation of the phase‐separating inks affected the final material porosity in gels prepared by casting the polymer solution into molds, followed by crosslinking at a uniform cytocompatible polymerization intensity (*I* = 5 mW cm^−2^; λ = 405 nm). The final material porosity depended on the concentration of both dextran and HA. The size of the final material porosity scaled proportionally with dextran concentration from 8.7 ± 2.2 to 19.4 ± 3.9 µm, at 0 and 5 wt% dextran respectively (Figure S3, Supporting Information) and decreased with an increasing concentration of HA from 23.2 ± 1.6 to 7.2 ± 1.3 µm at 0.05 wt% and to 0.3 wt% HA (Figure S4, Supporting Information). We next demonstrated that we could spatially control pore size by modulating the polymerization kinetics. Given that kinetics can be governed by the irradiation conditions for thiol–ene photopolymerizations, this was achieved by varying the irradiation intensity without changing dextran or HA concentration (**Figure** **2**a).^[^ ^18^, ^19^, ^22^ ^]^ Increasing the irradiation intensity decreased the time to reach complete gelation in our GelNb–PEG‐SH based bioinks, which was indicated by the time to reach plateau storage modulus (G’, Figure S5, Supporting Information). The polymerization kinetics determine the time to reach network percolation, which competes with the dynamics of the phase separation process. Extending the time until full percolation allowed the system to proceed further in the phase separation, leading to larger pore sizes and providing a handle to tune the final porosity in the material from a single formulation. We generated different porosities from a single formulation by polymerizing the hydrogels using different irradiation intensities under light emitting diode (LED) light (Figure 2b). We used the BoneJ plugin in Fiji to quantify the porosity in both 2D and 3D that featured pore segmentation, skeletonization, and determination of the pore widths (Figure 2c,d).^[^ ^17^, ^18^ ^]^ Decreasing the irradiation intensity from 10 to 0.5 mW cm^−2^ generated materials with pores ranging from 13.0 ± 2.4 to 52.9 ± 18.2 µm, respectively (Figure 2e). While the porosity fraction remained largely consistent across the range of polymerization intensities, a minor reduction was noted at the highest intensity (10 mW cm^−^^2^) that was significant in comparison with the medium polymerization intensity (5.6 mW cm^−^^2^). The fraction of porosity was 0.38 ± 0.1 for 10 mW cm^−^^2^ and 0.51 ± 0.04 for 5.6 mW cm^−^^2^ intensities (Figure 2f).

These unique properties of the phase‐separating bioresins enable new directions in their use in VBP, which operates with locally controllable light fields, wherein phase separation can be used to form objects with locally controlled micron‐scale porosity. In this manner, we could form objects with different intrinsic porosity through printing objects at different light intensity or objects with porosity gradients, by applying the light fields with gradient intensity (Figure 2g). We demonstrated this by volumetrically printing objects at different irradiation intensities (Figure 2h). We printed the objects with intrinsic porosities ranging between 17.5 ± 3.2 µm and 8.3 ± 2.0 µm in a single print volume, polymerizing the objects at different average irradiation intensities (*I*_av_) of 2 and 10 mW cm^−2^, respectively (Figure 2i). The difference between casted and printed porosity might stem from the light intensity distribution across the print volume during the volumetric printing and potential differences in oxygen concentrations during crosslinking (Figure S6, Supporting Information).

Although there were differences in pore sizes between the casted gels and printed gels at equivalent irradiation intensities, the scaling of pore size with intensity was conserved. While the process allows for control over the average irradiation intensity across the construct, localized regions can experience peak intensities up to 4–8 times higher than the average (depending on settings and the printed architecture), potentially influencing phase separation dynamics and pore formation. We further used a custom‐built VBP module to generate a hierarchical porosity within a single construct. By applying a volumetric light projections with gradient irradiation intensity linearly decreasing from *I_av_* = 8 to 0.8 mW cm^−2^ along the z‐axis we printed a cylindrical construct with a continuous porosity gradient increasing from 14.7 ± 1.6 µm to 67.7 ± 12.5 µm (Figure 2j).

### 2.3 Vascularization of Macroporous Printed Constructs

The porosity of phase separating hydrogels has been shown to be permissive for the cell infiltration, migration, and outgrowth of multicellular structures.^[^ ^14^, ^19^ ^]^ We hypothesized that the interconnected pore space within our hydrogels would be permissive for the outgrowth of the vascular structures potentially vascularizing volumetrically printed constructs with different sizes and architectures. To enable the formation of microcapillary‐like structures with diameters below 10 µm within volumetrically printed constructs, we precisely modulated the printing intensity (*I_av_* = 8–10 mW cm^−^^2^) to control phase separation dynamics during printing. Using this approach we fabricated constructs with intrinsic porosity below 10 µm, that would limit the outgrowth of larger vessels and favor development of microvascular networks within the printed architectures. To demonstrate pore permissiveness at scale relevant for mammalian cells we infused the pore space of the printed hydrogels with green fluorescent microbeads (d = 6 µm) that infiltrated in the pores of the construct (Figure S7, Supporting Information).

**Figure 3:**
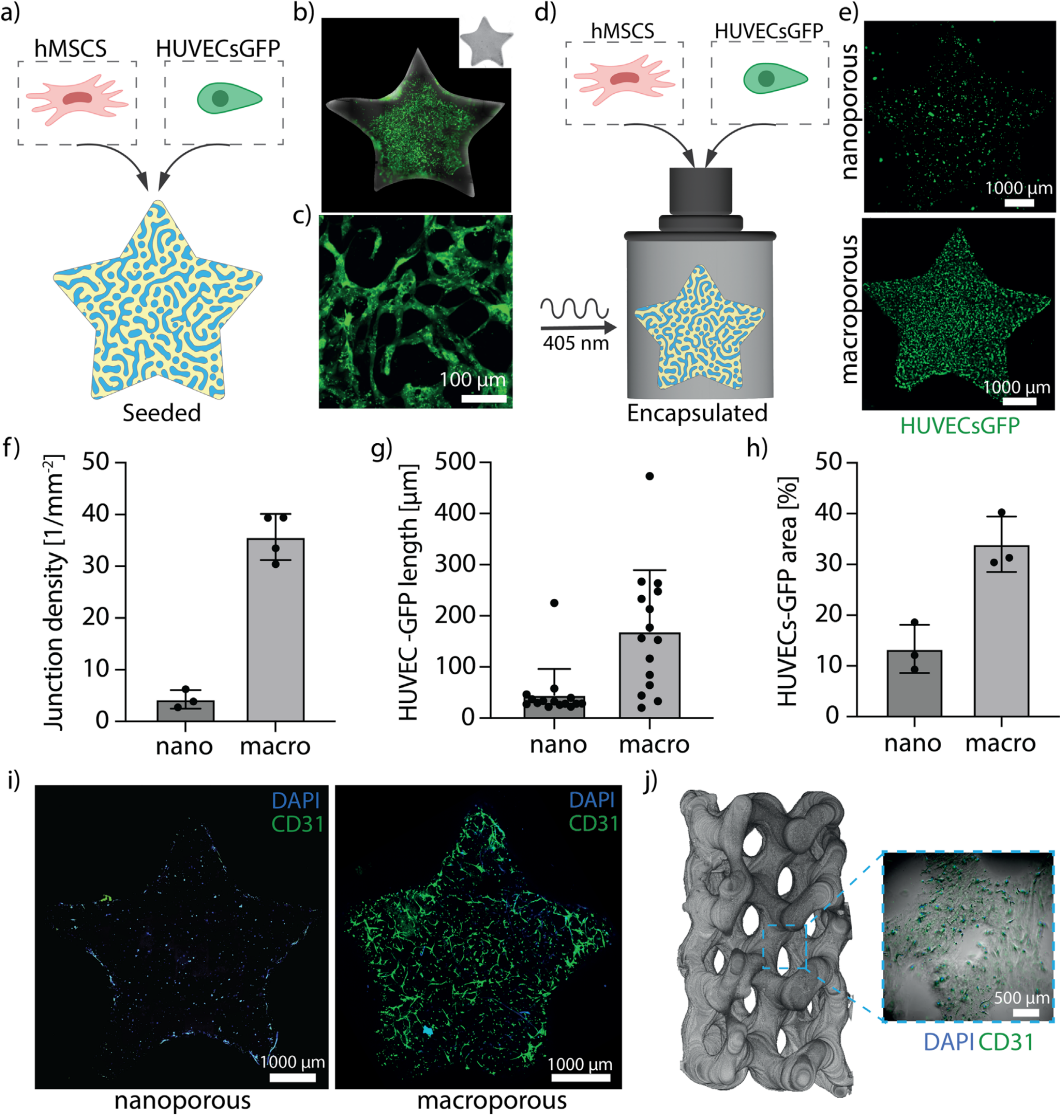
Macroporosity enhances vascular network formation in bioprinted constructs. a) The volumetrically printed shapes were seeded with the HUVECs‐GFP and hMSC. b) The formed endothelial network partially vascularized the porous space of the printed constructs showing limited ingrowth into material bulk. c) Endothelial structures in the porous space of the seeded hydrogel constructs. d) To improve vascularization of the printed construct the phase‐separating gels were volumetrically printed with cells suspended in gel forming solution. e) The printed constructs with nano‐ and macroporosity exhibited different cell infiltration and outgrowth of HUVECs‐GFP. f) The total junction density was 4.3 ± 1.8 mm^−2^ in nano‐ and 37.4 ± 3.4 mm^−2^ in macroporous constructs (*N* = 3). g) The average vessel length was 33.6 ± 3.0 in nanoporous and 169.7 ± 30.9 µm in macroporous printed constructs (*N* = 3). h) The total cell area was 13.4 ± 4.8 in nano and 33.99 ± 5.46% in macroporous constructs (*N* = 3). All values in f‐h are reported as mean and std. dev. i) Confocal tile scan of cell‐laden bioprinted stars with nano‐ and macroporosity. In nanoporous prints HUVECs‐GFP appeared mostly rounded. HUVECs‐GFP organized into the CD31 positive nascent vascular structures within the macroporous construct with an average diameter of 8.2 ± 2.7 µm. j) Light‐sheet reconstruction of the gyroid with multi‐scale porosity laden with hMSCs and HUVECs‐GFP (1:1, 500*10^3^ cells/ml), that developed vascular structures in the macroporous space (cropped image).

We first mechanically characterized different compositions of nanoporous and macroporous materials measuring Young's moduli, stain at break and energy at break at different weight percentages. We selected 3wt% nano‐ and macroporous inks with Young's moduli in the soft tissue regime (4–6 kPa) for further cell bioprinting experiments (Figures S8–S10, Supporting Information).^[^ ^20^ ^]^ To generate vasculature within the macroporous space of our printed constructs, we seeded GFP‐expressing human umbilical vein endothelial cells (HUVECs‐GFP) in combination with human mesenchymal stem cells (hMSCs) on top of the printed structures (**Figure** **3**a). The seeded cells cooperatively evolved into vascular‐like networks on the surface of the printed constructs and within the pore space with a mean diameter of 13.2 ± 5.7 µm over the period of 3 to 5 days. These endothelial networks grew into the pore space to depths of ≈300 µm but did not populate the whole volume of the prints, partially due to an uneven distribution of the cells on the surface of the constructs during seeding (Figure 3b,c; Figure S11, Supporting Information). To improve population of the porous space, we volumetrically printed the constructs with cells suspended in the gel forming bioink (*I_av_* = 8–10 mW cm^−^^2^) evenly distributing the cells in the in situ phase separating gels (Figure 3d). We fabricated the constructs both with nano‐ and macroporosity to assess if micron‐scale porosity improves vascular outgrowth in 3D space compared to the nanoporous hydrogels. Cell viability assessed 24 h after printing was comparable between the two groups, 73.8 ± 5.7% in nanoporous hydrogels and 71.3 ± 17.9% in macroporous hydrogels (Figure S12, Supporting Information). After 5 days of culture, we analyzed the morphology and outgrowth of HUVECs‐GFP within nano‐ and macroporous hydrogels. GFP‐positive cells within the nanoporous hydrogels were rounded, while macroporous prints exhibited more spreading and organization (Figure 3e). The endothelial networks developed throughout the entire volume of the macroporous printed constructs (Figure S13, Supporting Information).

The average vessel length was 33.6 ± 3.0 in nanoporous and 169.7 ± 30.9 µm in macroporous printed constructs (Figure 3g). The total cell area percentage taken up by HUVECs‐GFP was 13.4 ± 4.8% in nano‐ and 33.9 ± 5.5% in macroporous constructs (Figure 3h). The total junction density of vascular structures was 4.3 ± 1.8 mm^−2^ in nano‐ and 37.4 ± 3.4 mm^−2^ in macroporous constructs (Figure 3f). The nanoporous cell‐laden prints exhibited limited to no cell spreading, except for the cells attached to the outer rim of the printed construct, while endothelial structures positive for CD31 have formed over a period of 5 days in macroporous constructs (Figure 3i). The endothelial structures within macroporous stars had an average vessel diameter of 8.2 ± 2.7 µm. Interestingly, over time some of the cell‐laden macroporous prints exhibited shrinking while retaining the formed vascular structures (Figure S14, Supporting Information). The shrinking was most prominent in smaller and thinner constructs and at longer culture times. Similar behavior is reported for natural matrices (e.g., collagen) and likely reflects contractility‐driven matrix remodeling, highlighting productive engagement between encapsulated cells and the surrounding matrix.^[^ ^21^ ^]^ We also demonstrated formation of vascular structures in the larger constructs with multi‐scale porosity by printing large gyroids with cells (Figure 3j), to underline the versatility of printing complex architectures in which cells can grow.

### 2.4 Vascularization of Perfused Bioprinted Chips

Finally, we generated perfusable systems to test if perfusion enhances the vascular outgrowth and increases the stability of the formed vascular structures. These systems hold potential to be used in combination with other cell types, spheroids or organoids to generate perfusable vascularized organ or tumor models. Earlier, researchers have demonstrated guidance of angiogenesis from larger vessels using cell‐instructive cues, including growth factors, and mechanical stimulation using fluid flow.^[^ ^22^, ^23^, ^24^ ^]^ However, achieving vascularization of large perfusable constructs based on synthetic hydrogels remains a significant and unresolved challenge.

**Figure 4:**
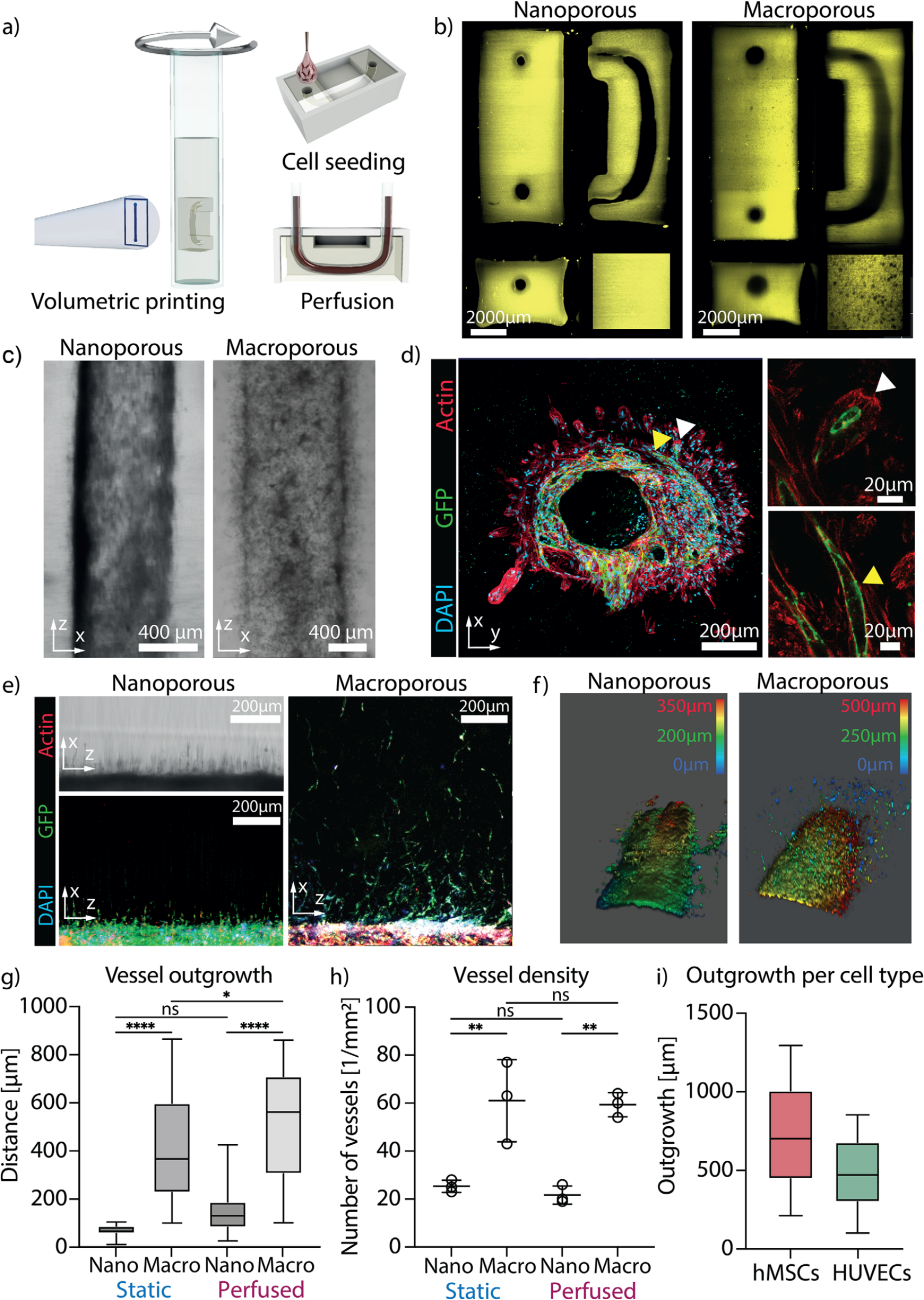
Vascularization of the printed perfusable constructs. a) Perfusable constructs volumetrically printed, with a central perfusable channel that was seeded with the hMSCs and HUVECs. b) Light‐sheet reconstruction of the perfusable chips generated with nano‐ and macroporosity. c) After one week of pre‐culture the cells in the nanoporous chips have shown limited outgrowth into the bulk of the construct; the macroporous chips exhibited improved cell infiltration from the channel. d) Left: The seeded hMSCs and HUVECs infiltrated the bulk of the constructs from the main channel, the hMSCs were followed by HUVECs; Top‐right, bottom‐right: hMSC‐driven protrusions that are lined with HUVECs and lumenized. e) The macroporous chips (right) show enhanced vascularization compared to the chips with nanoporosity (left) along the length of the perfusable channel. The outgrowth in the nanoporous constructs is mainly realized through printing striations as shown by transmitted light micrograph (top left). f) 3D reconstruction of confocal z‐stacks (500 µm) of the main channel in the nano‐ and macroporous perfused constructs showing enhanced outgrowth of the cells in macroporosity. g) Vascular outgrowth within nano‐ and macroporous constructs (*N* = 3). h) The vessel density in nano and macroporous constructs kept under static conditions was 25.3 ± 2.5 mm^−2^ and 61.0 ± 17.09 mm^−2^ respectively. In perfused constructs the vessel density was 21.7 ± 2.8 mm^−2^ and 59.3 ± 5.0 mm^−2^ for the nano‐ and macroporous construct respectively (*N* = 3). i) Analysis of cell‐specific outgrowth showed that on average hMSCs migrated over longer distances from the main channel than HUVECs (*N* = 3). The migration distances were 735.8 ± 330.8 µm for hMSCs and 472.0 ± 220.2 µm for HUVECs‐GFP. Aligned dot plot represents mean and st. dev. (** = p < 0.01, ns = not significant; one‐way ANOVA). Box plots represent the interquartile range (25th–75th percentile), medians, and whiskers show the full data range (minimum to maximum;* = p < 0.05, ** = p < 0.01, **** = p < 0.0001; ns = not significant; one‐way or two‐way ANOVA).

We volumetrically printed constructs with a large perfusable central channel (*d* ≈ 800 µm) both with nano‐ and macroporosity (**Figure** **4**a). In constructs printed with phase‐separating bioinks, interconnected porosity extended into the bulk of the material from the central channel. As a control, nanoporous constructs were printed using GelNb–PEG‐SH without the excluding components, dextran and HA (Figure 4b). Although the material bulk was nanoporous, orthogonal striations have formed along the main channel, a common artefact of the volumetric printing. The channel of the constructs, designed with a u‐shaped curvature to promote retention of the seeded cells, was seeded with the HUVECs‐GFP and hMSCs (1:1 ratio; 10 µL, total of 1 × 10^6^ cells). Following seeding, the chips were rotated around their longitudinal axis for 6 h using a custom‐built rotating device to ensure uniform distribution of cells along the channel walls. The seeding was repeated twice over a one‐week pre‐culture period. Subsequently, a subset of the constructs was maintained under static culture conditions, while the remainder was subjected to perfusion culture in a custom‐designed bioreactor system (Figure S15, Supporting Information).^[^ ^25^ ^]^ The constructs were perfused with a micropump for the period of 1 week at constant flow rates starting at 300 µL mi^(1^n for the period of two days to limit cell detachment, following with increased flow rates of 700 µL mi^(1^n until the end of perfusion. These flow rates resulted in the shear stresses along the channel wall ranging from 0.8 to 2 dyn cm^−2^.^[^ ^26^ ^]^ This range of shear stress was hypothesized to improve stability of the main vessel lining without restriction of angiogenic sprouting.^[^ ^27^, ^28^ ^]^

Within the macroporous constructs the cells grew into the bulk of the material within the first week of pre‐culture; the cells were mostly allocated within the channel in the nanoporous constructs showing limited outgrowth into the striations (Figure 4c). The striations that were formed during the printing process create narrow, anisotropic channels and enabled cell invasion into otherwise non‐permissive nanoporous materials.^[^ ^25^ ^]^ In the macroporous constructs, the hMSCs infiltrated porous space starting from the central channel spanning distances of up to 250 µm within the first 5 days of culture. Interestingly, even though HUVECs‐GFP and hMSCs were seeded together they self‐organized into an inner layer of endothelial cells (green) surrounded by an outer layer of hMSCs (red) (Figure 4d). This arrangement recapitulates the native vascular architecture, where endothelial cells are surrounded by stromal perivascular cells, stabilizing the vessel architecture.^[^ ^29^, ^30^ ^]^ HUVECs‐GFP had not completely lined the protrusions led by hMSCs but cross sections show partly lumenized endothelial linings in some of the protrusions.

After 2 weeks of culture, macroporous constructs maintained under static and perfused conditions exhibited visibly outgrown vascular structures extending into the bulk of the material. Some of these structures exhibited hierarchical branching (Figure S16, Supporting Information). The porous constructs maintained under static conditions exhibited cell outgrowth over the distances of 405.0 ± 239.9 µm. The perfused macroporous constructs facilitated evolution of vascular structures spanning over the distances of 520.4 ± 224.0 µm. There was also a limited outgrowth in the nanoporous constructs over the distances of 64.5 ± 29.1 µm and 147.4 ± 83.3, for static and perfused conditions respectively (Figure 4g; Figure S17, Supporting Information). The outgrowth in nanoporous constructs was mainly allocated within striated regions with locally reduced crosslinking density.^[^ ^25^ ^]^ Overall, the macroporosity significantly improved vascularization of the materials bulk compared to nanoporous conditions. Under nanoporous conditions, perfusion did not yield a statistically significant gain in outgrowth, likely due to the relatively high stiffness and lack of cell‐level porosity of the material, although perfused samples showed a trend toward deeper cell infiltration. In contrast, perfusion significantly enhanced vascular outgrowth in macroporous gels. Taken together, these results suggest that perfusion promotes vascular invasion when pore size is permissive, though the effect is variable and needs further investigation.

The vessel density was higher in the macroporous compared to nanoporous constructs in both static and perfused conditions. In static conditions the vessel density was 25.3 ± 2.5 mm^−2^ and 61.0 ± 17.1 mm^−2^ for the nano‐ and macroporous construct respectively. In perfused constructs the vessel density was 21.7 ± 2.8 mm^−2^ and 59.3 ± 5.0 mm^−2^ for the nano‐ and macroporous construct respectively (Figure 4h). Interestingly, while outgrowth distance seemed to be affected by perfusion, vessel density remained largely unaffected. This could be due to the fact that the vessel density is usually regulated through angiogenic growth factors (e.g., VEGF) signalling rather than by the shear forces initiated by perfusion.^[^ ^31^, ^32^ ^]^

Within the perfused macroporous chip the migration over the distances of 735.8 ± 330.8 µm for hMSCs and 472.0 ± 220.2 µm for HUVECs‐GFP was observed, with hMSCs migrating over longer distances from the main channel than HUVECs‐GFP (Figure 4i). The longer distances (>500 µm) were mainly reached through the migration of hMSCs rather than vascular outgrowth. This is likely due to a higher migratory capacity of the hMSCs and their ability to individually navigate porous space, whereas HUVECs typically rely on collective sprouting or need strong angiogenic cues for migration.

## 3 Conclusion

With vascularization being a major challenge in the biofabrication of viable and functional living tissues, there is a clear need for materials that support dynamic cellular processes such as spreading, migration, and tissue infiltration. In this study, we demonstrated that phase‐separating photopolymerizable bioinks, when combined with volumetric 3D bioprinting, enable the fabrication of constructs with controllable features on multiple scales – large millimeter‐scale porosity derived from the printing process and micrometer‐scale porosity arising from phase separation. Furthermore, the use of volumetric light fields not only allows for rapid fabrication but also offers precise control over the extent of phase separation and resulting porosity through modulation of light intensity enabling formation of porosity gradients within the printed structures. This hierarchical pore architecture is particularly well‐suited for promoting vascularization in large (centimeter‐scale) free‐form bioprinted structures. Importantly, the formation of interconnected porous networks addresses the inherent limitations of synthetic hydrogels in supporting cell infiltration and tissue integration. This synergy between light‐controlled fabrication and material behavior presents a powerful strategy for engineering functional vascularized tissues. Although the method permits tuning of porosity size and architecture, vascular outgrowth remains partly random, governed by the available porosity and intrinsic constraints on vessel spacing and distribution. Spatial control over microvascular network organization therefore remains fundamentally limited, and achieving this precision will require advanced biofunctionalization strategies such as incorporating growth factors or engineered biochemical gradients to move from porosity‐driven guidance toward predictable vascular network formation.^[^ ^33^ ^]^ Moreover, vascular guidance could also be achieved implementing new technologies that can detect regions of interest within the materials, and tune the printed tissue geometry locally, to direct cell migration and infiltration.^[^ ^34^ ^]^

The generated perfusable constructs with intrinsic porosities enabled infiltration of vascular structures deep into the bulk of hydrogels. The main objective of the present study focused on establishing the structural and biological feasibility of the material platform to demonstrate that the resulting porosity is permissive for multicellular outgrowth, with particular emphasis on the capacity of the porosity to support sprouting of vascular networks. Our data also shows early hMSC and endothelial cell network commitment using established vascular markers, such as αSMA and CD31. It should be noted that bone‐derived hMSC natively lack or show low expression of αSMA, which is instead upregulated during pericytic differentiation,^[^ ^35^ ^]^ though αSMA is also found to be expressed as an artefact of 2D expansion on tissue culture plastic, something that can be mitigated using cells at low passages.^[^ ^36^ ^]^ Future studies should investigate longer‐term cellular fate, and its dependency with gradual exposure to increasing level of flow‐induced mechanical stresses, and more comprehensive maturation markers (i.e., nitric oxide production, von Willebrand factor, ZO‐1, PDGFRβ, among others). Further, the hMSCs exhibited greater migratory capacity than HUVECs, especially over longer distances (>500 µm), where cell presence was predominantly attributable to hMSC migration, consistent with their high motility. These results highlight the need for additional guidance cues, such as gradients of angiogenic factors, optimization of perfusion parameters and mechanical stimuli (e.g., peristaltic flow), and prolonged perfusion times to enhance directed vascular outgrowth over even longer distances. The VBP platform is highly compatible with the integration of such spatiotemporal biochemical and physical signals, offering a versatile strategy for engineering functional, vascularized tissue and organ‐on‐chip systems.^[^ ^33^ ^]^ This approach is well‐suited for generating perfusable, macroporous tissue‐ or organ‐on‐chip platforms potentially combining them with organoids or spheroids of the target tissue.

## 4 Experimental Section

### Hydrogel Production

Hydrogel forming solution for making nanoporous gels were obtained by mixing the following components at final concentrations of 3% w/v GelNb (BioInx), 0.05% w/v LAP (Lithium phenyl(2,4,6‐trimethylbenzoyl)phosphinate, Tokyo Chemical Industry, Japan), 0.75% w/v PEG‐SH. The hydrogel mix was always made on the same day as it was used and components were weighed in sterile conditions or sterile filtered for use with cells. The mix was kept in the dark at 37 °C and vortexed for at least 10 s before being used.

Hydrogel forming solutions for making macroporous gels were obtained by mixing the following components at final concentrations of 3% w/v GelNb (BioInx), 0.05% w/v LAP (Lithium phenyl(2,4,6‐trimethylbenzoyl)phosphinate, Tokyo Chemical Industry, Japan), 0.75% w/v PEG‐SH, 0.1% w/v HA, 3% w/v dextran. The hydrogel mix was always made on the same day as it was used and components were weighed in sterile conditions or sterile filtered for use with cells. The mix was kept in the dark at 37 °C and vortexed for at least 10 s before being used.

### Rheological Characterization

The cross‐linking kinetics and mechanical properties of the GelNB/PEG‐SH hydrogels (3–5 wt%) were quantified using a discovery hybrid rheometer (DHR; TA Instruments). The hydrogel precursor solution was loaded between a 25 mm parallel plate geometry (PP‐08) and a transparent bottom plate with a gap of 0.5 mm. The gel forming solution was cross‐linked using collimated UV light (λ = 365 nm, *I* = 20 mW cm^−2^; Bluepoint). Storage (*G*′) and loss (*G*″) moduli were measured over time with oscillatory strain measurements at γ = 1% amplitude and with an angular frequency of ω = 1 Hz (Figure S8, Supporting Information). All measurements were carried out in the linear viscoelastic regime. Young's modulus (*E*) was estimated using the relation between shear and Young's moduli for isotropic and homogeneous materials, *E* = 2*G*′(1+ν) where ν is the Poisson's ratio for the material. For the materials tested, ν was assumed to be 0.5.

### Mechanical Characterization

Hydrogel precursor solutions were poured into cylindrical molds (6 mm diameter, 2 mm height) and irradiated with light from a 405 nm LED at 6 and 10 mW cm^−2^ for 120 and 180 s respectively. After gelation, the hydrogels were incubated in phosphate‐buffered saline (PBS) at 37 °C overnight. Mechanical tests were conducted using a dynamic mechanical analyzer (DMA Q850, TA Instruments). In compressive mechanical testing, samples were compressed with a preload force of 0.001 N at a constant strain rate of 20% per minute until reaching a 30% strain. The compressive modulus was obtained from the linear region of the stress‐strain curve. Another set of samples was subjected to a force ramp from the preload force 0.001 N to 18 N at a rate of 1 N per minute until failure. The energy at breaking was calculated as the area under the force‐displacement curve.

### Cell Culture

hMSCs were isolated from iliac crest bone marrow aspirates obtained from consenting patients (one donor, female; approved by the research ethics committee of the University Medical Center Utrecht, isolation 08–001, distribution protocol 18–739) were cultured in MSC expansion medium (10% FCS, 1% pen/strep, 1% asap in alpha‐MEM (Gibco)) supplemented with 5 ng mL^−1^ bFGF (PeproTech) on 0.1% w/v gelatin coated cell culture flasks and medium was refreshed twice a week. MSCs were split at a ratio of 1:3 once a week.

GFP‐HUVECs were cultured in Endothelial cell growth medium‐2 (EGM‐2 MV Microvascular Endothelial Cell Growth Medium‐2 BulletKit; Lonza; CC‐3202) on 0.1% w/v gelatin coated cell culture flasks and medium was refreshed twice a week. HUVECs were split at a ratio of 1:3 once a week. For cell experiments, passages between 3 to 9 and 6 to 10, for hMSCs and HUVECs‐GFP, respectively, were used in this study.

### Light Intensity Versus Porosity

15 µL droplets of hydrogel mix were pipetted onto a PDMS surface. Droplets were exposed to 2.5, 5.6 or 10 mW light at 405 nm for 5 min. Droplets were transferred into a well plate well containing 8 µL/mL Cy3.5 in PBS0 and stored at 4 °C overnight to take up the dye. The next day, samples were washed with PBS0 and submerged in dextran‐FITC solution for at least 1 h before imaging using the Leica Stellaris confocal microscope. Z‐stacks of 50 µm size were acquired to assess homogeneity of pores throughout the gel.

### Analysis of Porosity and Pore Sizes

The 3D micrograph image stack was processed within ImageJ. First binary thresholding was performed to segment the regions corresponding to the voids (pores) and gel. Porosity was determined by dividing the void volume by the total volume of the imaged sample. To determine the 3D pore sizes, the image stacks were further processed using the plugin BoneJ, enabling skeletonization of the voids, and subsequent determination of pore size distributions within the samples.^[^ ^17^, ^18^ ^]^

### Cell Seeding or Encapsulation in Constructs Printed Via Volumetric Bioprinting

For volumetric printing, hydrogel mix was pipetted into 10 mm glass vials, vortexed and kept at 37 °C before printing. Additionally, nanoporous conditions were prepared by excluding dextran and HA from the hydrogel mix. Printing was performed with a Tomolite 1.0 bioprinter (Readily3D) and Apparite software. A refractive index of 1.37 and a peak‐to‐average ratio of 4:1 were set, and printing occurred with 6 mW cm^−2^ average intensity and 55–60 mJ cm^−2^ light dose. Post printing, hydrogels were washed thoroughly with warm PBS0. To seed the hydrogels, a cell suspension of 15 uL with a 1:1 hMSCs:GFP‐HUVECs ratio for a total of 1 million cells was pipetted on top of the hydrogels. For cell encapsulation studies, a cell pellet containing a 1:1 hMSCs:GFP‐HUVECs ratio at 0.5 million cells per mL was resuspended in the hydrogel mix prior to printing, and, post printing, the hydrogels were washed with warm medium. Live/dead staining with the viability/cytotoxicity dyes calcein‐AM and ethidium homodimer‐1 was performed on days 1 and 3 after printing. The medium was changed every 2 to 3 days, and the hydrogels were imaged with a Thunder Leica microscope.

### Volumetric Bioprinting, Cell Seeding and Perfusion of Vessel on‐a‐Chip Models

Volumetric printing of vessel on‐a‐chip constructs was executed as previously described.^[^ ^25^ ^]^ Briefly, 1 mL of 37 °C hydrogel mix was filled into 10 mm glass vials. Chip models were loaded into apparite software in vertical orientation. Chips were printed at a light dose of 50–70 mJ/cm^2. Chips were washed thoroughly and channels flushed with warm PBS0 and each channel was controlled for perfusability by injecting warm PBS0 into the channel. Chips were glued to well plate wells using 10% w/v PEGDA with 0.1% w/v LAP and seeded with MSCs and HUVECs at a 1:1 ratio for a total of 1 million cells. This cell suspension was done in a 7.5% w/v gelatin solution in medium as the increased viscosity facilitates seeding. After cell injection chips were rotated by 90 degrees along their longitudinal axis every 10 min for 6 h using a custom‐built rotating device. Warm 1:1 hMSC:EGM‐2 medium was added to the site but not the top of the chips to prevent flushing out the cells. The seeding was repeated twice over a one‐week pre‐culture period. Then, chips were transferred into a custom‐designed perfusion bioreactor system and perfusion was initiated at 300 µL mi^(1^n for 2 days, and at 700 µL mi^(1^n until the end of culture. Shear stress, dependent on channel diameter (D), flow rate (Q) and fluid viscosity (η), was calculated according to Equation 1.^[^ ^26^ ^]^ With channel diameter (D) ≈ 850 µm, flow rate (Q) = [300–700] µL mi^(1^n, and assuming approximately (η) ≈ 0.001 Pa·s for cell medium viscosity at physiological conditions, these flow rates correspond to shear stress values of 0.8 and 1.9 dyn/cm^2^, respectively.

(1) $$\tau =\eta 32Q/\pi D_{3}$$

For analyzing the cell infiltration in the perfusion experiments, for each experimental condition, the experiments were run in triplicate (*N* = 3), a field of view in the middle of the construct was imaged, and vessel sprouts/migrating line of cells were measured in each individual sample depending on what observed in the field of view, with each field selected to have least seven measurable sprouts per sample. Sprouts that were not fully included in the field of view (i.e., cut out at the border of the image) were not counted.

### Volumetric Bioprinting of Hierarchical Porosities

Three cylinders of the same size were inserted into Apparite software and stacked on top of each other. Cylinders were printed in sequential sessions using light intensities of 35 and 350 mJ cm^−2^ respectively. Cylinders were washed with warm PBS0 and cut into coronal cross sections to assess hierarchical porosities under the bright‐field microscope.

### Sample Preparation, Immunofluorescence Stainings and Confocal Imaging

Samples were prepared as previously described.^[34]^ Briefly, samples were fixed for 1.5 h at 4 °C in 4% w/v ice cold PFA and subsequently washed with PBT (PBS + 0.1% w/v Tween20) for at least 30 min (or stored in PBT until further processing). Using a vibratome, samples were cut into 350 µm slices. Samples were washed in wash buffer 2 (WB2, 1 L PBS + 1 mL of Triton X‐100 + 2 mL of 10% (w/v) SDS + 2 g BSA) for 30 min at 4 °C. Samples were stained for CD31 (1:200; Biolegend; 102 513), F‐actin (1:200; SigmaAldrich 79286‐10NMOL) and DAPI (1:1000; Thermo Fisher Scientific Invitrogen D1306) overnight at 4 °C. Subsequently, samples were washed three times for 1.5 h in WB2, mounted onto thin glass slides and imaged using the Leica Stellaris or SP8 confocal microscope using a 10, 20 and 25× water immersion objectives.

### Statistical Analysis

The experiments were designed and performed with at least three replicates for each condition, and statistical analysis was conducted using Prism 10 (GraphPad). Conditions were assessed using one‐way analysis of variance (ANOVA), with Šídák's multiple comparisons test, or two‐ way ANOVA. *P* values less than 0.05 were considered significant.

## Conflict of Interest

The authors declare no conflict of interest.

## Author Contributions

The project was conceived and designed by O.Y.D., R.L., and M.‐B.B., and O.Y.D., R.L., M.‐B.B., and G.G. designed experiments. O.Y.D., M.‐B.B., G.G., S.A., S.F., A.R.F. carried out the experiments and analyzed data. O.Y.D., R.L. M.‐B.B., G.G., M.W.T. wrote the manuscript. All authors have approved the final version of the manuscript.

## Supporting information



Supporting Information

## Data Availability

The data that support the findings of this study are available from the corresponding author upon reasonable request.

## References

[1] D. Dai , J. Fernandes , X. Sun , L. Lupton , V. Payne , A. Berk , Journal of Health Economics and Outcomes Research 2024, 11, 75. 38523709 10.36469/001c.94710 PMC10961141

[2] Mater. Des. 2023, 229, 111885.

[3] T. Kaully , K. Kaufman‐Francis , A. Lesman , S. Levenberg , Tissue Eng Part B Rev 2009, 15, 159. 19309238 10.1089/ten.teb.2008.0193

[4] W. J. Polacheck , M. L. Kutys , J. B. Tefft , C. S. Chen , Nat. Protoc. 2019, 14, 1425. 30953042 10.1038/s41596-019-0144-8 PMC7046311

[5] S. Sundaram , J. H. Lee , I. M. Bjørge , C. Michas , S. Kim , A. Lammers , J. F. Mano , J. Eyckmans , A. E. White , C. S. Chen , Nature 2024, 636, 361. 39663490 10.1038/s41586-024-08175-5 PMC12235753

[6] K. Bott , Z. Upton , K. Schrobback , M. Ehrbar , J. A. Hubbell , M. P. Lutolf , S. C. Rizzi , Biomaterials 2010, 31, 8454. 20684983 10.1016/j.biomaterials.2010.07.046

[7] Z. Wei , M. Lei , Y. Wang , Y. Xie , X. Xie , D. Lan , Y. Jia , J. Liu , Y. Ma , B. Cheng , S. Gerecht , F. Xu , Nat. Commun. 2023, 14, 1. 38097553 10.1038/s41467-023-43768-0 PMC10721650

[8] E. Baur , M. Hirsch , E. Amstad , Adv. Mater. Technol. 2023, 8, 2201763.

[9] F. Vanlauwe , C. Dermaux , S. Shamieva , S. Vermeiren , S. Van Vlierberghe , P. Blondeel , Front. Bioeng. Biotechnol. 2024, 12, 1452477. 39380897 10.3389/fbioe.2024.1452477 PMC11458444

[10] G. L. Ying , N. Jiang , S. Maharjan , Y.‐X. Yin , R. R. Chai , X. Cao , J. Z. Yang , A. K. Miri , S. Hassan , Y. S. Zhang , Adv. Mater. 2018, 30, 1805460. 10.1002/adma.201805460 PMC6402588 30345555

[11] G. Ying , N. Jiang , C. Parra‐Cantu , G. Tang , J. Zhang , H. Wang , S. Chen , N. P. Huang , J. Xie , Y. S. Zhang , Adv. Funct. Mater. 2020, 30. 10.1002/adfm.202003740 PMC7941201 33708030

[12] P. N. Bernal , M. Bouwmeester , J. Madrid‐Wolff , M. Falandt , S. Florczak , N. G. Rodriguez , Y. Li , G. Größbacher , R. A. Samsom , M. van Wolferen , L. J. W. van der Laan , P. Delrot , D. Loterie , J. Malda , C. Moser , B. Spee , R. Levato , Adv. Mater. 2022, 34, 2110054. 10.1002/adma.202110054 35166410

[13] B. Grigoryan , S. J. Paulsen , D. C. Corbett , D. W. Sazer , C. L. Fortin , A. J. Zaita , P. T. Greenfield , N. J. Calafat , J. P. Gounley , A. H. Ta , F. Johansson , A. Randles , J. E. Rosenkrantz , J. D. Louis‐Rosenberg , P. A. Galie , K. R. Stevens , J. S. Miller , Science 2019, 364, 458. 31048486 10.1126/science.aav9750 PMC7769170

[14] O. Y. Dudaryeva , L. Cousin , L. Krajnovic , G. Gröbli , V. Sapkota , L. Ritter , D. Deshmukh , Y. Cui , R. W. Style , R. Levato , C. Labouesse , M. W. Tibbitt , Adv. Mater. 2025, 37, 2410452. 39745118 10.1002/adma.202410452 PMC11837887

[15] M. Z. Müller , et al., Nat. Commun. 2025, 16, 4923. 40425560 10.1038/s41467-025-60113-9 PMC12116776

[16] S. G. Lévesque , R. M. Lim , M. S. Shoichet , Biomaterials 2005, 26, 7436. 16023718 10.1016/j.biomaterials.2005.05.054

[17] M. Doube , M. M. Klosowski , I. Arganda‐Carreras , F. P. Cordelières , R. P. Dougherty , J. S. Jackson , B. Schmid , J. R. Hutchinson , S. J. Shefelbine , Bone 2010, 47, 1076. 20817052 10.1016/j.bone.2010.08.023 PMC3193171

[18] R. Domander , A. A. Felder , M. Doube , Wellcome Open Res 2021, 6, 37. 33954267 10.12688/wellcomeopenres.16619.1 PMC8063517

[19] N. Broguiere , A. Husch , G. Palazzolo , F. Bradke , S. Madduri , M. Zenobi‐Wong , Biomaterials 2019, 200, 56. 30772759 10.1016/j.biomaterials.2019.01.047

[20] C. T. McKee , J. A. Last , P. Russell , C. J. Murphy , Tissue Eng Part B Rev 2011, 17, 155. 21303220 10.1089/ten.teb.2010.0520 PMC3099446

[21] A. Ding , K. L. Gasvoda , D. S. Cleveland , S. Ayyagari , E. Alsberg , Adv Sci. 2025, 07288. 10.1002/advs.202507288 PMC12752645 41001773

[22] D. Guo , Q. Wang , C. Li , Y. Wang , X. Chen , Oncotarget 2017, 8, 77020. 29100366 10.18632/oncotarget.20331 PMC5652760

[23] D.‐H. T. Nguyen , S. C. Stapleton , M. T. Yang , S. S. Cha , C. K. Choi , P. A. Galie , C. S. Chen , Proc Natl Acad Sci 2013, 110, 6712. 23569284 10.1073/pnas.1221526110 PMC3637738

[24] P. A. Galie , D.‐H. T. Nguyen , C. K. Choi , D. M. Cohen , P. A. Janmey , C. S. Chen , Proc. Natl. Acad. Sci. USA 2014, 111, 7968. 24843171 10.1073/pnas.1310842111 PMC4050561

[25] M. B. Buchholz , P. N. Bernal , N. Bessler , C. Bonhomme , R. Levato , A. Rios , Biofabrication 2025, 17, 031001. 10.1088/1758-5090/add20f 40300616

[26] E. Roux , P. Bougaran , P. Dufourcq , T. Couffinhal , Front Physiol 2020, 11, 861. 32848833 10.3389/fphys.2020.00861 PMC7396610

[27] V. K. Lee , A. M. Lanzi , H. Ngo , S. S. Yoo , P. A. Vincent , G. Dai , Cell Mol. Bioeng. 2014, 7, 460. 25484989 10.1007/s12195-014-0340-0 PMC4251565

[28] V. K. Lee , D. Y. Kim , H. Ngo , Y. Lee , L. Seo , S. S. Yoo , P. A. Vincent , G. Dai , Biomaterials 2014, 35, 8092. 24965886 10.1016/j.biomaterials.2014.05.083 PMC4112057

[29] G. Yang , B. Mahadik , J. Y. Choi , J. P. Fisher , Progress in biomedical engineering 2020, 2, 012002. 34308105 10.1088/2516-1091/ab5637 PMC8302186

[30] K. Pill , J. Melke , S. Mühleder , M. Pultar , S. Rohringer , E. Priglinger , H. R. Redl , S. Hofmann , W. Holnthoner , Front Bioeng Biotechnol 2018, 6, 156. 30410879 10.3389/fbioe.2018.00156 PMC6209673

[31] E. L. Lund , C. Thorsen , M. W. Pedersen , N. Junker , P. E. Kristjansen , Clin. Cancer Res. 2000, 6, 4287. 11106245

[32] S. Ylä‐Herttuala , T. T. Rissanen , I. Vajanto , J. Hartikainen , J Am Coll Cardiol 2007, 49, 1015. 17349880 10.1016/j.jacc.2006.09.053

[33] M. Falandt , et al., Adv. Mater. Technol. 2023, 8. 10.1002/admt.202300026 PMC7615165 37811162

[34] S. Florczak , G. Größbacher , D. Ribezzi , A. Longoni , M. Gueye , E. Grandidier , J. Malda , R. Levato , Nature 2025, 645, 108. 40903601 10.1038/s41586-025-09436-7 PMC12408377

[35] K. Joensuu , L. Uusitalo‐Kylmälä , T. A. Hentunen , T. J. Heino , J Tissue Eng Reg Med 2018, 12, 775. 10.1002/term.2496 28593699

[36] I. Aizman , W. S. Holland , C. Yang , D. Bates , Cell Med. 2016, 8, 79. 28003933 10.3727/215517916X693357 PMC5165647

