# Supplementary material for: Multi‐scale Engineered Vasculature and Hierarchical Porosity via Volumetric Bioprinting‐Guided Photopolymerization‐Induced Phase Separation
Source: Adv Mater. 2025 Dec 9;38(9):e21171. doi: 10.1002/adma.202521171 (PMC12902586; doi:10.1002/adma.202521171)
Supplement: Supplementary file 1 — Supporting Information [file ADMA-38-e21171-s001.docx]

**Supplementary Information for:**

**Multi-scale Engineered Vasculature and Hierarchical Porosity via Volumetric Bioprinting-guided Photopolymerization-induced Phase Separation**

*Oksana Y. Dudaryeva, Maj-Britt Buchholz, Gabriel Größbacher, Sofia Amaral, Sammy Florczak, Camille Bonhomme, Alvaro Rojo Ferrer, Mark W. Tibbitt, and Riccardo Levato**

*Correspondence to : [r.levato@uu.nl](mailto:r.levato@uu.nl)

**Supporting Information**


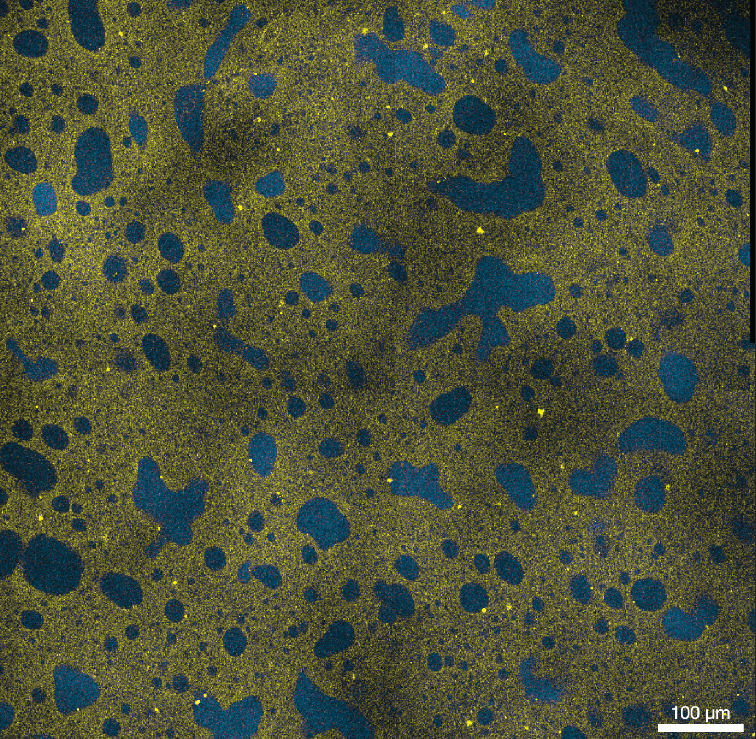


**Figure S1.** Irregular porosity in GelNb–PEG–SH hydrogels without temperature control. In the absence of temperature regulation during hydrogel cross linking, the GelNb–PEG–SH system forms irregular, binodal structures instead of the desired bicontinuous porosity. The gel phase appears in yellow, while the pore space is visualized in blue using high-molecular-weight Dextran-FITC (500 kDa).


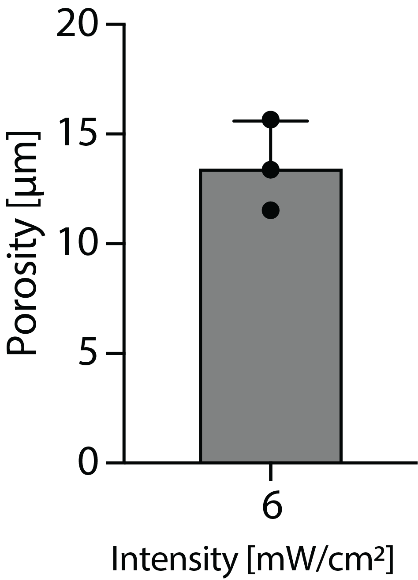


**Figure S2.** Pore size in gyroid constructs printed at I = 6 mW cm² (n = 3, 3 measurements per replicate). Experimental values are presented as mean ± standard deviation (SD).


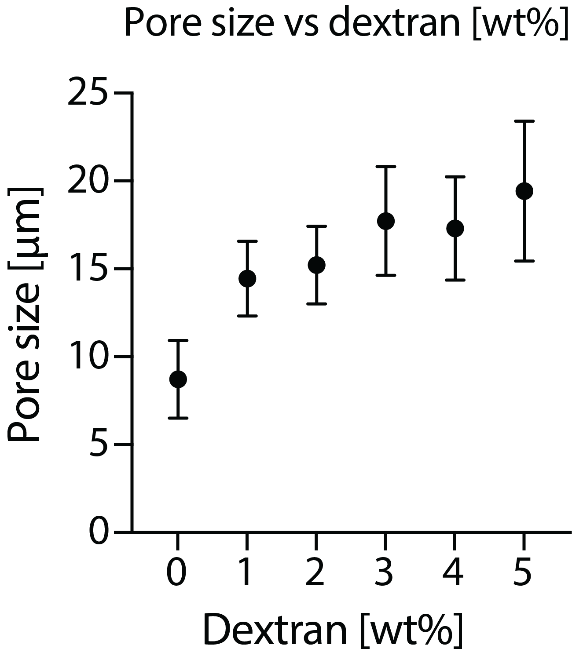


**Figure S3.** Pore size in 3 wt% Gel-Nb–PEG–SH hydrogels polymerized under constant light intensity (I = 5 mW cm²; n = 3) increased at increasing dextran concentration (from 0 to 5 wt%). Pore size increased with dextran content with a statistically significant difference among mean pore sizes across dextran levels (Welch’s ANOVA, p < 0.0001). Experimental values are presented as mean ± standard deviation (SD).


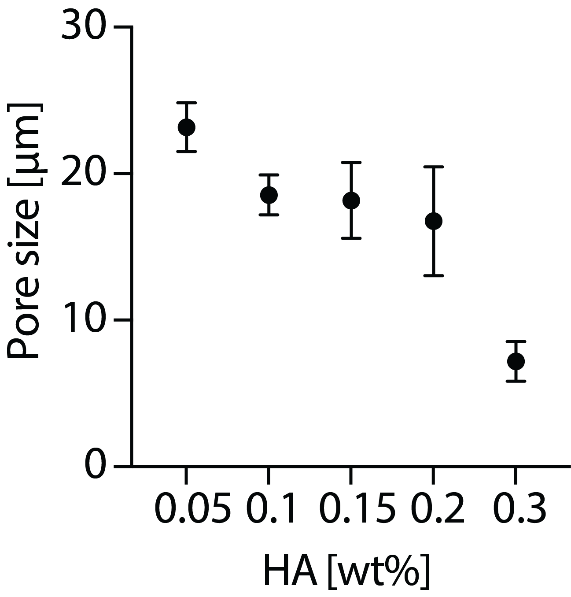


**Figure S4.** Pore size in 3 wt% Gel-Nb–PEG–SH hydrogels polymerized under constant light intensity (I = 5 mW cm²; n = 3) decreased at increasing HA (2 MDa) concentration (from 0.05 to 0.3 wt%). Ordinary one-way ANOVA showed a significant effect of HA content on pore size (p<0.0001; R²=0.78). Experimental values are presented as mean ± standard deviation (SD).


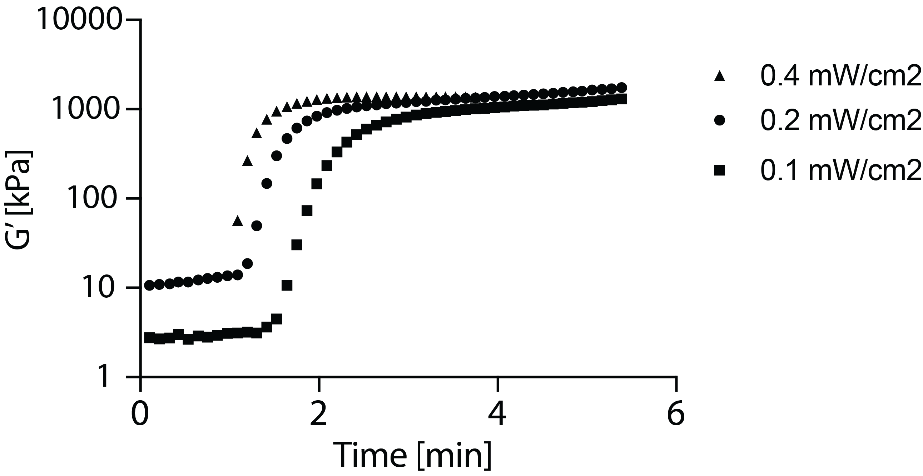


**Figure S5. Influence of irradiation intensity on gelation kinetics in Gel-NB–PEG–SH hydrogels.** The evolution of the storage modulus (G') during photopolymerization was monitored using a rheometer to assess gelation kinetics in macroporous Gel-NB–PEG–SH hydrogels (3 wt%) exposed to varying light intensities. All samples were irradiated starting at the same time point (1 minute after measurement onset). Lower light intensities led to slower increases in G' and delayed plateau formation, indicating that crosslinking kinetics are strongly dependent on irradiation intensity. The hydrogels crosslinked at different light intensities reach the same plateau modulus.


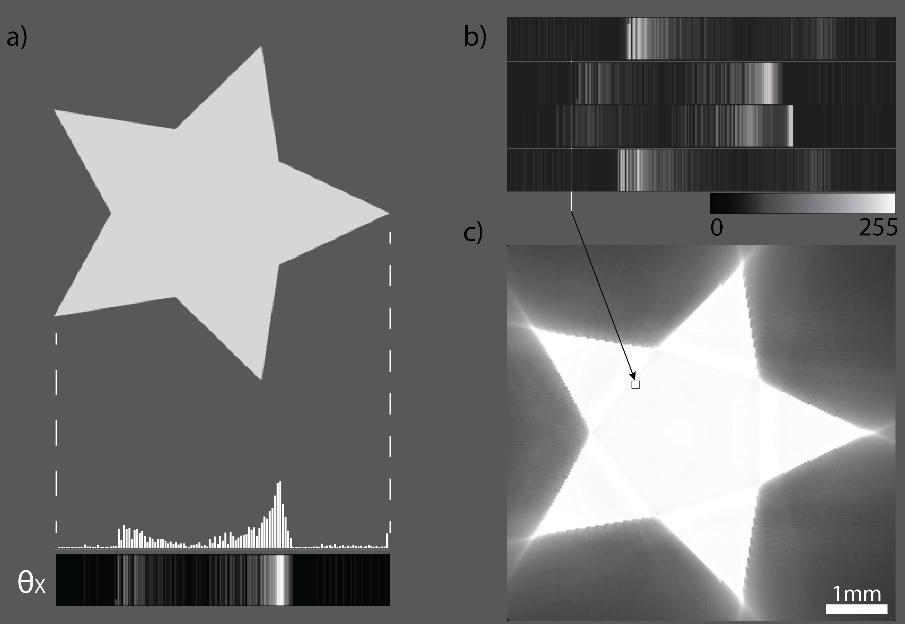


**Figure S6**. Intensity distribution during volumetric printing. a) An example of a projected pattern at an arbitrary angle (θx) for the tomographic projection of a star, demonstrating the non-homogeneous intensity distribution at each projected angle. b) Four example projections each generated at a different projection angle, in which a 2D pixel is targeting the same voxel. While the projected pixels vary in light intensity at each projected angle, the sum of all pixels reaches the photocrosslinking threshold within the object's voxel (arrow targets a hypothetical voxel volume). In contrast, absolute grayscale values are linked to an absolute light intensity. c) The filtered tomographic back projection of the printed star, showing the final reconstructed light dose distribution.


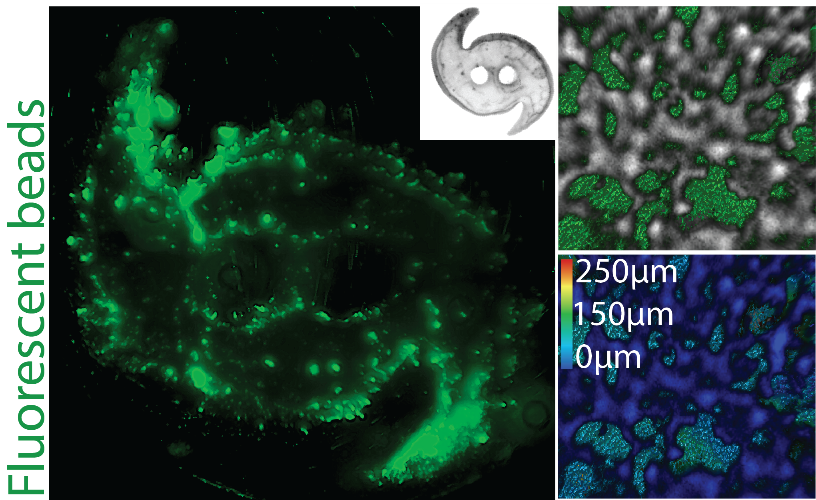


**Figure S7**. Maximum projection of the macroporous galaxy print that was agitated with the green fluorescent microbeads (d = 6 μm) with the insert of the corresponding printed shape. The interconnected porous space allowed the penetration of microbeads into the construct (left). The 3D reconstruction of the porous space shows the accumulation of the microbeads (green) within the pores of the hydrogel (gray) to the depths of 150 μm.


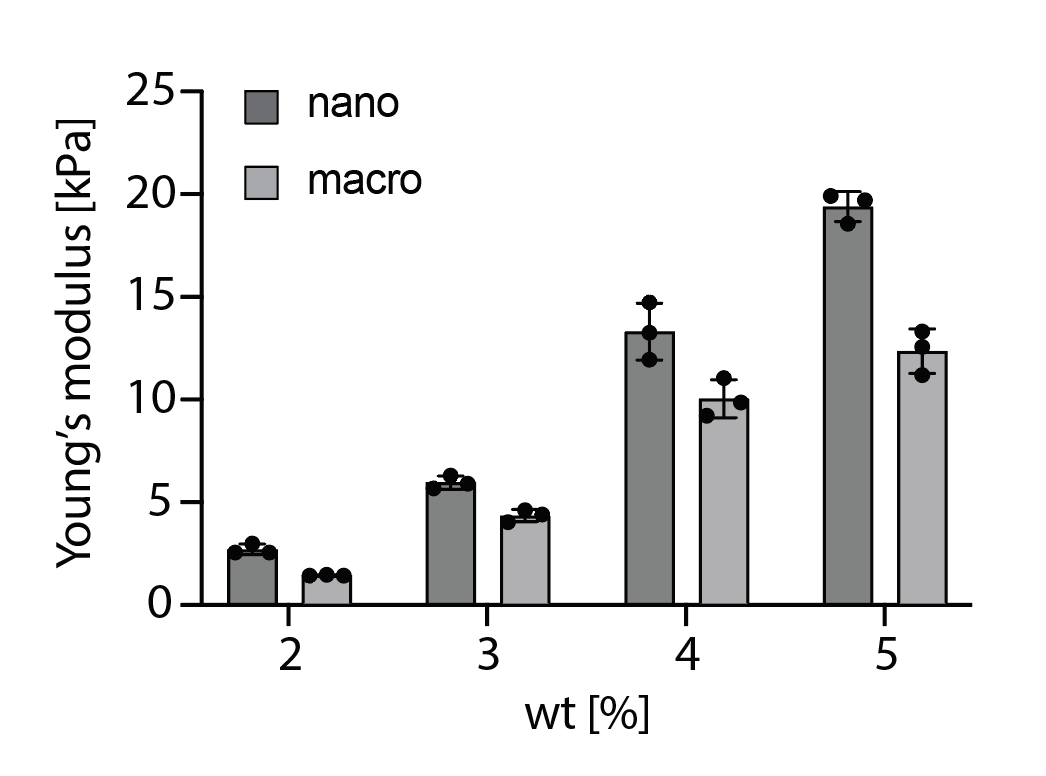


******

*****

******

*****

**Figure S8**. Young’s modulus (E) of nano- and macroporous GelNB hydrogels as a function of polymer content (2–5 wt%). Bars show mean E (kPa), circles are individual replicates (n = 3 per condition), and whiskers indicate SD. The hydrogels at 3wt% were selected with the mean moduli of 5.9 ± 0.3 kPa for nanoporous hydrogels and 4.3 ± 0.3 kPa for macroporous hydrogels. Bar graph represents mean and st. dev., * = p < 0.05, ** = p < 0.01; unpaired t-test, nanoporous vs. microporous.


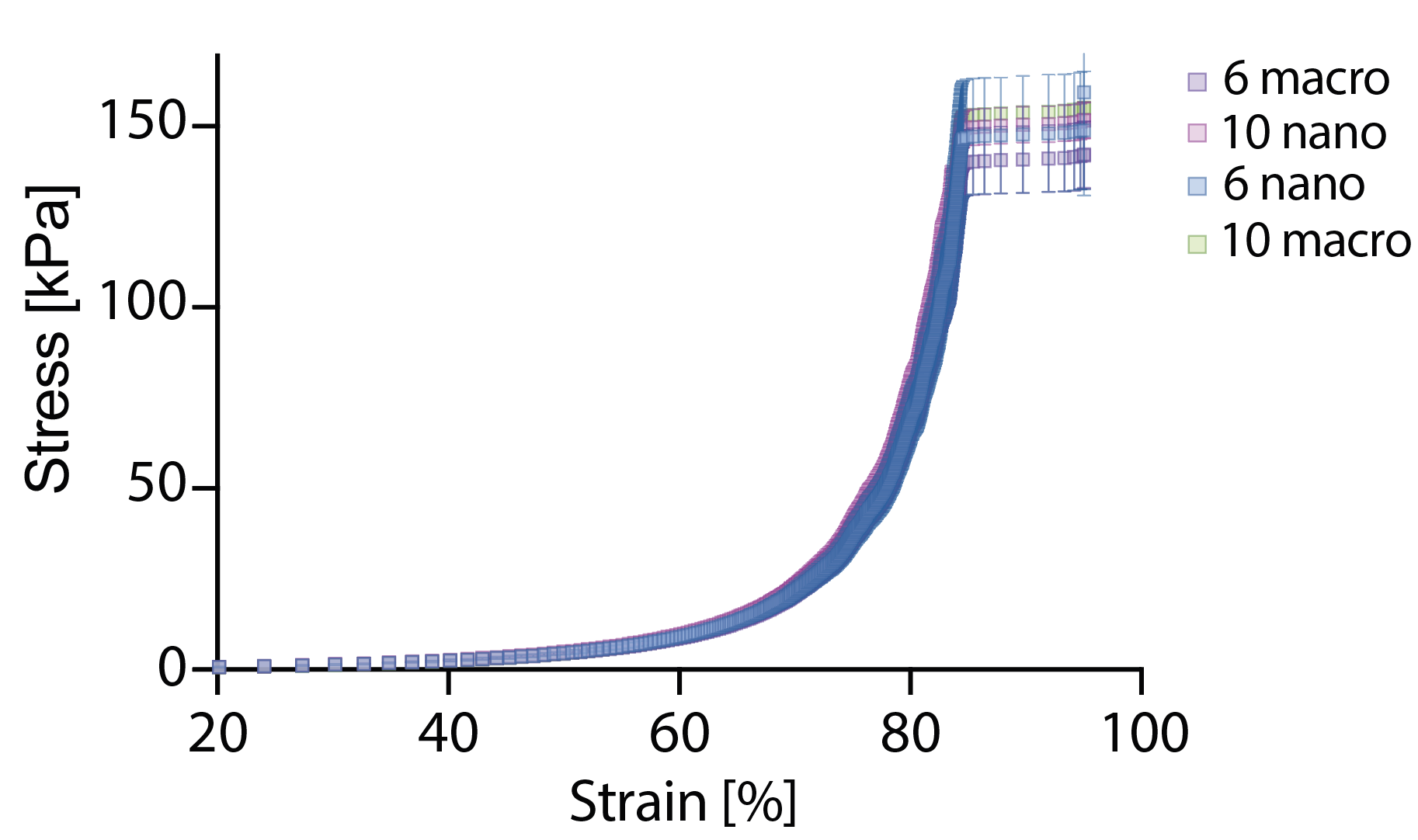


**Figure S9**. Compressive stress–strain curves for 3 wt% GelNB hydrogels with nano- or macroporous architectures, photocrosslinked at 6 or 10 mW/cm^2^ (n = 3 per condition). All formulations failed at ε ≈ 0.83–0.84 (≈ 84% compressive strain). Ultimate stress at break (σᵤ, mean ± SD) for the nanoporous gels crosslinked at 6 mW/cm² was 132.7 ± 14.5 kPa and at 10 mW/cm² was 136.9 ± 4.5 kPa; and for the macroporous gels crosslinked at 6 mW/cm² was 118.6 ± 15.0 kPa, and at 10 mW/cm² was 138.5 ± 28.6 kPa. Nanoporous gels reached slightly higher σᵤ. Both formulations showed increase in σᵤ at higher crosslinking intensity. Ultimate stress at break (σᵤ, mean ± SD) for the nanoporous gels crosslinked at 6 mW/cm² was 132.7 ± 14.5 kPa and at 10 mW/cm² was 136.9 ± 4.5 kPa; and for the macroporous gels crosslinked at 6 mW/cm² was 118.6 ± 15.0 kPa, and at 10 mW/cm² was 138.5 ± 28.6 kPa.


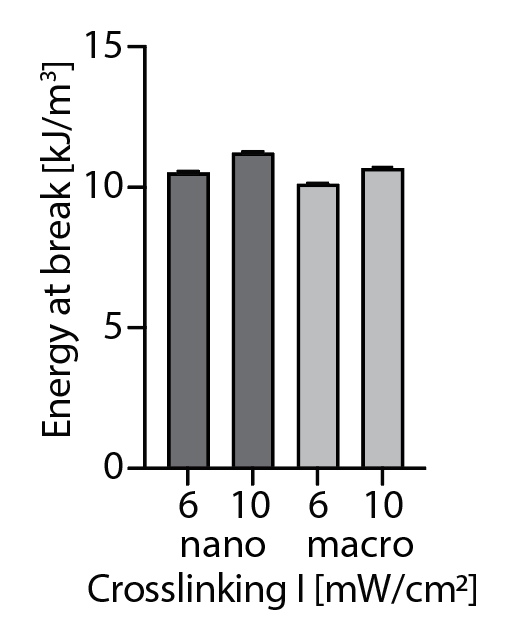


**Figure S10**. Energy at break (compressive toughness calculated as area under the stress–strain curve to failure) for 3 wt% GelNB hydrogels with nano- or macroporosity, photocrosslinked at 6 or 10 mW/cm² (n = 3). Energy at break for nanoporous gels crosslinked at 6 mW/cm² was 10.56 ± 0.04 kJ/m³ and at 10 mW/cm² was 11.27 ± 0.03 kJ/m³. Energy at break for the macroporous gels crosslinked at 6 mW/cm² was 10.16 ± 0.02 kJ/m³ and at 10 mW/cm² was 10.80 ± 0.01 kJ/m³. Nano gels absorbed slightly more energy before failure than macro gels and light intensity had only a minor effect. Bars show mean ± SD.


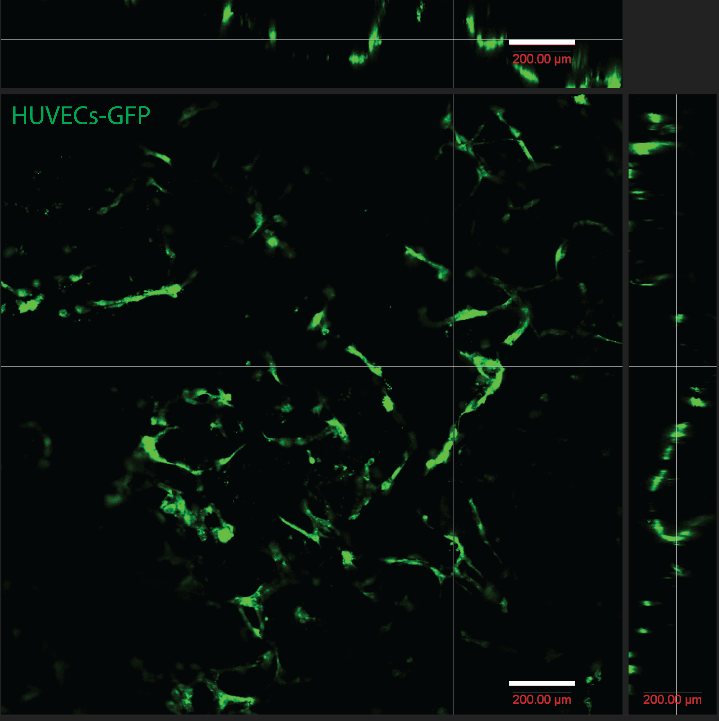


**Figure S11. Infiltration of HUVECs-GFP into porous hydrogels.** HUVECs-GFP were seeded onto the surface of porous hydrogel constructs. Wishing 5 day of the culture period, the cells actively migrated and infiltrated into the pore space, reaching depths of up to 300 μm. Imaging was performed using confocal microscopy to visualize and quantify the depth of cellular penetration within the scaffold.


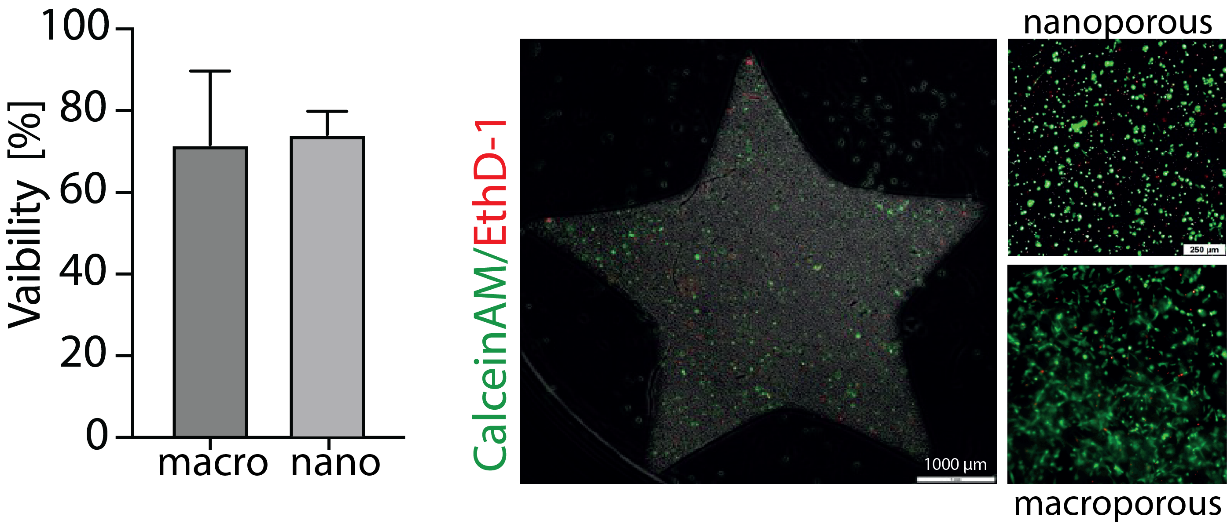


**Figure S12. Viability of printed cells within star-shaped constructs.** The viability of HUVECs-GFP and hMSCs encapsulated in star-shaped constructs printed under varying light intensities (4–10 mW/cm²) was assessed 24 hours post-printing. Cell viability was quantified using live/dead (Calcein-AM/Ethidium Homodimer-1) staining and fluorescence imaging. In nanoporous hydrogels, viability was 73.8 ± 5.7%, while in macroporous hydrogels it was 71.3 ± 18.0%, indicating comparable post-printing cell survival across both hydrogel architectures.


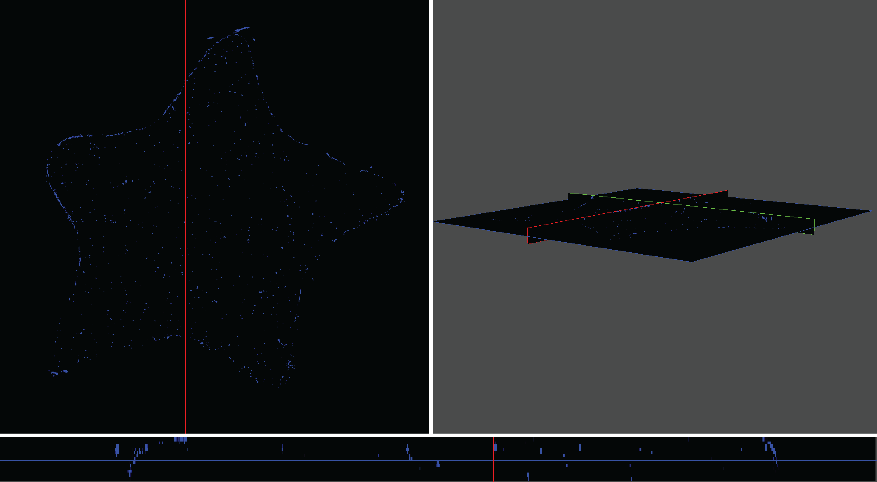


**Figure S13.** The distribution of DAPI signals within the volume of printed star constructs reconstructed from the z-stack of the confocal slices (total z-dimension length is 50 μm).


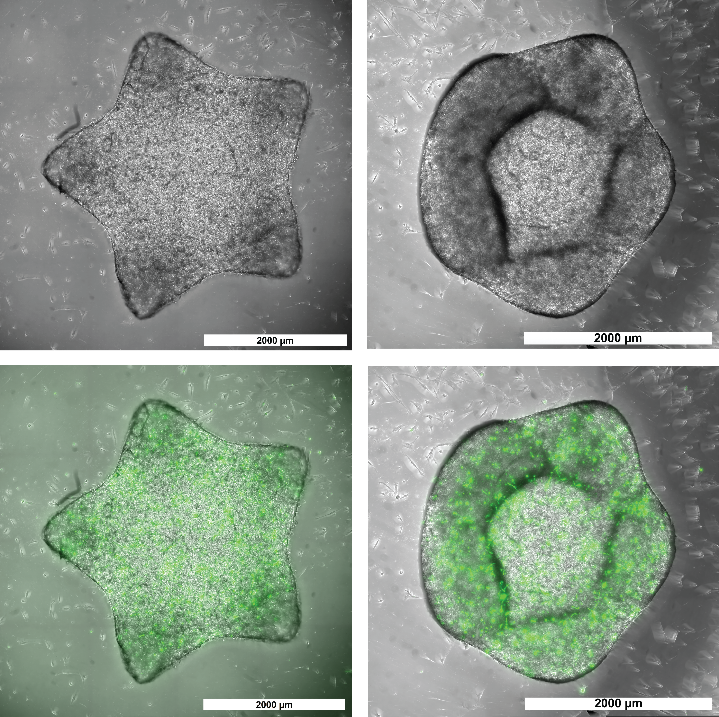


**Figure S14. Shrinking of macroporous star-shaped constructs.** Macroporous star-shaped constructs with a thickness of 0.5 mm supported the formation of endothelial structures throughout the hydrogel matrix. Over a 7-day culture period, some constructs exhibited noticeable shrinkage. Despite this macroscopic contraction, the internal endothelial networks remained structurally stable.


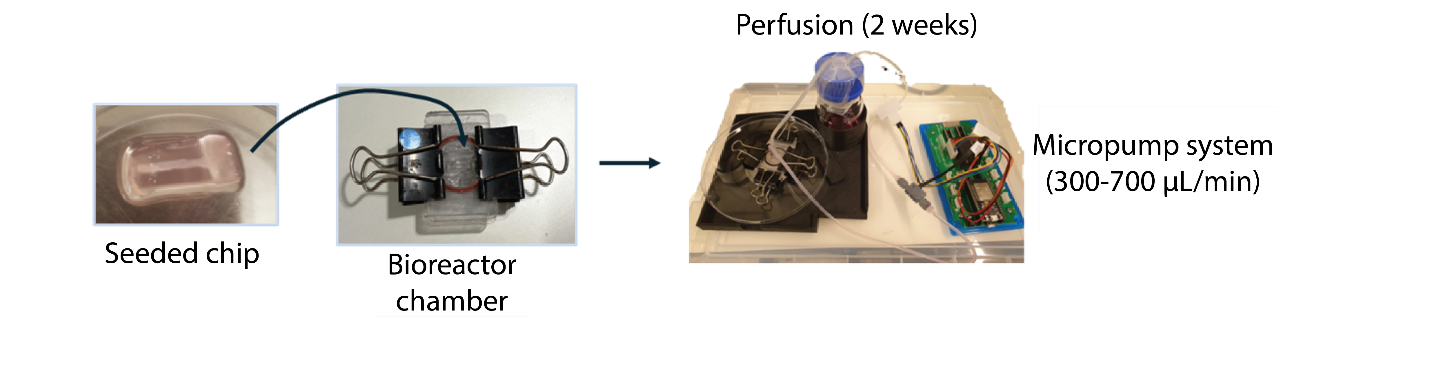


**Figure S15**. **Perfusion set-up.** The printed constructs that were seeded with HUVECs-GFP and hMSCs were placed in a bioreactor chamber equipped for connection to the perfusion tubing. The chamber was then connected to a micropump perfusion system, which continuously perfused the chips with fresh medium (EGM-2/alpha-MEM) to support HUVECs and hMSCs over a 2-week period under physiological conditions.


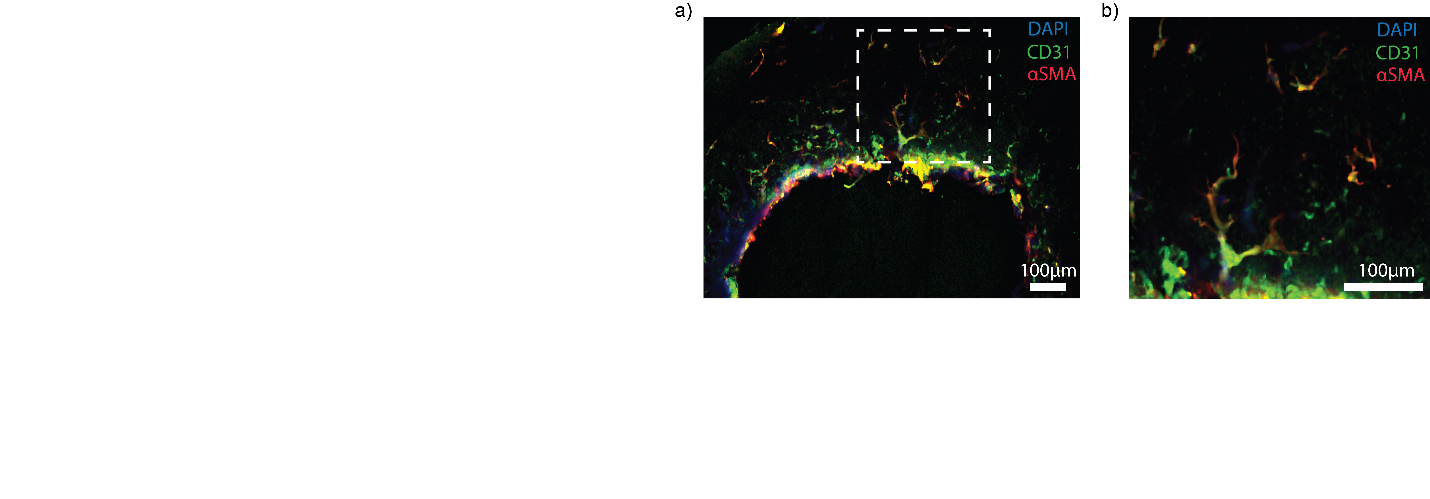


**Figure S16. Hierarchical vascular branching from the main perfused channel.** A radial cross-section of the main channel in the perfusable construct after 2 weeks of perfusion culture reveals the formation of organized, hierarchical vascular branches extending from the main channel into the surrounding porous hydrogel matrix.


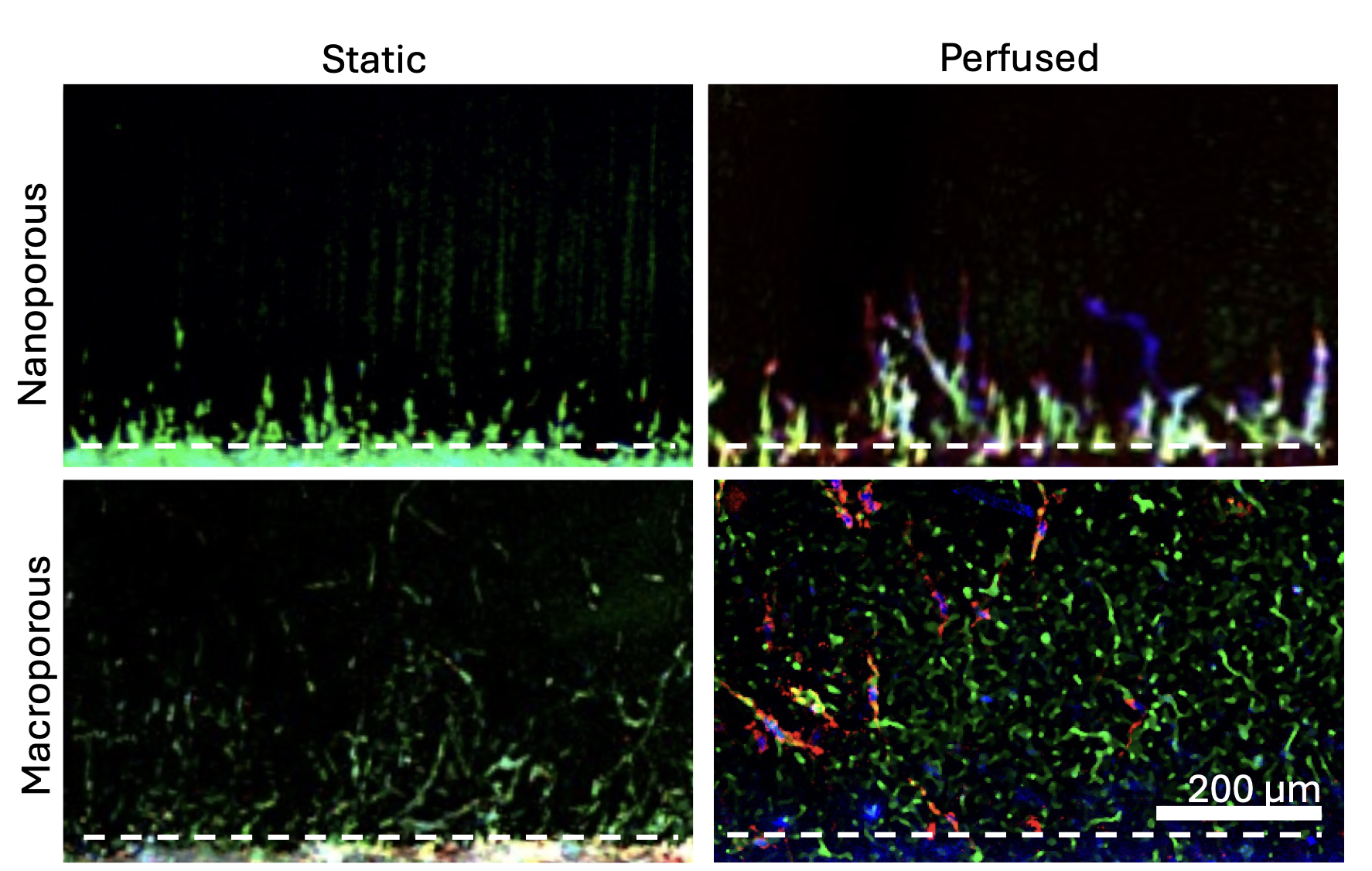


**Figure S17.** Comparison of vascular outgrowth in nanoporous and macroporous hydrogel constructs under static and perfused conditions. Confocal fluorescence images show endothelial network formation within nanoporous (top row) and macroporous (bottom row) hydrogels (DAPI - blue, CD31 - green, αSMA - red). Perfusion enhances vascular infiltration and network connectivity, particularly in macroporous scaffolds, where interconnected pores support extensive vessel formation throughout the matrix. Dashed lines indicate the onset of material bulk starting from the perfused vessel in the constructs. Scale bar: 200 µm.
